# Extended field-of-view two-photon imaging in the deep brain of freely behaving mice using lightweight microfabricated corrective optics

**DOI:** 10.1126/sciadv.aei5334

**Published:** 2026-10-16

**Authors:** Chiara Nardin, Andrea Sattin, Sebastiano Curreli, Giorgio Letti, Laura Maddalena, Leonardo Nacci, Tommaso Fellin

**Affiliations:** ^1^ Optical Approaches to Brain Function Laboratory, Istituto Italiano di Tecnologia, Genova, Italy.; ^2^ University of Genova, Genova, Italy.

## Abstract

Investigating neural circuits underlying complex behavior requires high-resolution imaging of large neuronal populations during naturalistic conditions. Miniaturized two-photon microscopes (MINI2Ps) enable deep brain imaging in freely moving animals when coupled with gradient index (GRIN)-based microendoscopes. However, intrinsic optical aberrations in GRIN-based microendoscopes degrade spatial resolution and limit the effective field-of-view (FOV). Here, we address these limitations using lightweight microfabricated optics to correct GRIN aberrations during deep brain MINI2P imaging. The aberration corrected system demonstrated improved and more homogeneous axial resolution across the FOV, resulting in more than fourfold increase in effective FOV. We applied the corrected system to the ventral posteromedial thalamus in freely moving mice and performed optical mapping of head direction (HD) tuned cells. These measurements revealed the absence of spatial clustering of HD preference, suggesting spatially sparse HD coding in sensory thalamus. Combining the MINI2P with aberration corrected GRIN-based microendoscopes is a powerful approach for large-scale and high-resolution functional imaging of deep brain circuits during naturalistic behavior.

## INTRODUCTION

Understanding how neural circuits generate behavior requires the ability to record activity from large populations of neurons with high spatial resolution in conditions that closely approximate natural behavior (*1*–*3*). Over the past decades, two-photon (2P) microscopy (*4*, *5*) combined with genetically encoded calcium indicators (*6*–*8*) has become a central tool for interrogating neural activity at cellular resolution. This approach has provided unprecedented insights into circuit function across multiple brain regions, revealing how neuronal populations encode sensory information (*9*–*11*), internal states (*12*–*14*), and motor-related signals (*14*–*16*). However, conventional 2P imaging has largely been restricted to head-fixed preparations (*17*, *18*), limiting its applicability to behaviors that require unrestricted movement and complex environmental interactions.

MINI2Ps have recently been developed to enable cellular-resolution imaging in freely moving animals (*19*–*22*). These systems represent a major technological advance, allowing the study of neural dynamics during naturalistic behaviors, such as foraging and navigation (*21*). To image deeper than 1.5–2.0 mm down the surface of the brain, MINI2Ps need to be coupled to endoscopic light guides, such as GRIN lenses (*23*). GRIN lens-based endoscopes relay the focal plane of the MINI2P objective to the target region of interest. This approach enables optical recordings in freely moving animals from previously inaccessible structures located several millimeters below the brain surface, including the thalamus, the hypothalamus, and the basal ganglia.

However, GRIN lens-based endoscopes have intrinsic optical aberrations, which severely degrade spatial resolution, especially in lateral portions of the FOV (*24*–*27*). These aberrations include spherical aberration, astigmatism, and coma, which become increasingly pronounced at off-axis positions (*25*, *26*). Aberrations of GRIN-lens based endoscopes alter the shape of the microscope point-spread function (PSF), degrade spatial resolution, and restrict the FOV (*25*, *26*, *28*, *29*). As a result, imaging quality is highly heterogeneous across the FOV, with peripheral regions often suffering from severe blurring and signal loss. These effects have detrimental consequences on functional imaging: reduced signal-to-noise ratio (SNR), increased cross-contamination between neighboring emitters, and the introduction of biases in the quantification of neuronal activity and pairwise correlations (*28*, *29*). Such biases are particularly problematic when studying neuronal populations, where uniform sampling across the imaging plane is essential for accurate characterization of population dynamics.

In this study, we leveraged on microfabricated optics to correct intrinsic aberrations in GRIN microendoscopes and integrated these corrected probes with MINI2P systems for deep brain imaging in freely moving mice. We systematically characterized the optical performance of corrected versus uncorrected systems, demonstrating increased spatial resolution, PSF homogeneity, and enlarged FOV. Moreover, we used this technology to investigate neuronal activity in the ventral posteromedial nucleus (VPM) of the thalamus, a deep sensory structure located more than 3 mm below the brain surface involved in the encoding of whisker kinematics and animals’ head direction during spatial navigation (*30*). Using corrected GRIN microendoscopes coupled to the MINI2P, we provided optical mapping of HD tuning in the mouse sensory thalamus, which highlighted the sparse organization of HD coding in the mouse VPM.

## RESULTS

To overcome the limitations introduced by optical aberrations of GRIN lens-based microendoscopes when using the MINI2P (*21*) for deep brain imaging, we used lightweight (weight < 0.5 mg) optics microfabricated with 2P lithography [3D printing (*31*)] and specifically designed to correct optical aberrations in GRIN microendoscopes [GRIN diameter: 500 μm; GRIN length: 4.1 mm or 6.4 mm (*28*, *29*)].

### Enlarged FOV imaging of MINI2P coupled with aberration corrected microendoscopes

We characterized the optical performance of the MINI2P alone, of the MINI2P combined with uncorrected GRIN microendoscopes, and of the MINI2P coupled to corrected GRIN microendoscopes. To this aim, we acquired serial optical sections along the axial direction (*z*-stacks) through: (i) subresolved homogeneous fluorescent layers (*32*), to visualize the axial extension of the excitation volume as a function of the distance from the center of the FOV (radial distance); (ii) fluorescent grids, to evaluate potential FOV distortion; (iii) subresolved fluorescent beads, to measure the radial and axial spatial resolution at different radial distances. The *x*,*z* projection of the *z*-stack acquired on the subresolved fluorescent layer with the MINI2P not coupled to a GRIN microendoscope was a flat stripe and displayed fairly homogeneous signal intensity over the radial distance (fig. S1A). Moreover, the MINI2P not coupled to a GRIN microendoscope showed FOV distortion (fig. S1B), which required calibration and post-hoc correction (*21*), and displayed homogeneous spatial resolution as a function of the radial distance (fig. S1, C and D).

To quantify the impact of microfabricated corrected optics on the optical performance of MINI2Ps when imaging through GRIN microendoscopes, we imaged the same samples using the MINI2P either coupled to uncorrected or to corrected GRIN microendoscopes (Fig. 1), initially using 4.1 mm-long GRIN microendoscopes. We set the focal distance of GRIN microendoscopes equal to the nominal focal length for which the aberration correction was designed [corresponding to *z*_i_ = 0 in the image space and *z*_s_ = 0 in the sample space in Fig. 1C, right inset; see also Materials and Methods and (*28*, *29*)]. We found that the *x*,*z* projection of the *z*-stack acquired on the subresolved fluorescent layer imaged with the MINI2P coupled to uncorrected microendoscopes (uncorrected system) was curved and displayed enlarged axial extension and decreased signal intensity in the lateral portions of the FOV (off-axis). These results were in agreement with the presence of several optical aberrations in GRIN microendoscopes (*28*), resulting in a restricted imaging FOV (Fig. 1D, top). In contrast, the transversal projection of the FOV of the MINI2P coupled to corrected microendoscopes (corrected system) displayed more regular thickness and signal intensity across radial distances, indicating efficacy in the correction of many optical aberrations and predicting an enlargement of the imaging FOV compared to the uncorrected system (Fig. 1D, bottom). As for the MINI2P alone, the combined optical system (MINI2P coupled with GRIN microendoscopes) displayed FOV distortion, which required calibration and post-hoc correction (fig. S2A). Importantly, using subresolved fluorescent beads we found that both in the center of the FOV (on-axis) and off-axis the corrected system displayed improved and more homogeneous axial resolution compared to the uncorrected system, in which instead the axial resolution rapidly degraded with the radial distance (Fig. 1, E and F, and table S1). Lateral resolution was instead comparable in the uncorrected and the corrected system (fig. S2B). To estimate the impact of aberration correction on the dimensions of the imaging FOV, we computed the radius of the effective FOV of each system based on a threshold applied on the axial resolution (see Materials and Methods for details). The enhanced and more homogeneous spatial resolution of the corrected system resulted in an expansion of the effective FOV area of more than four times with respect to the uncorrected system (table S2). Altogether, these initial set of experiments demonstrate that coupling the MINI2P with corrected GRIN microendoscopes enables imaging in substantially enlarged FOV and with more homogenous spatial resolution across the FOV.

**Fig. 1.**
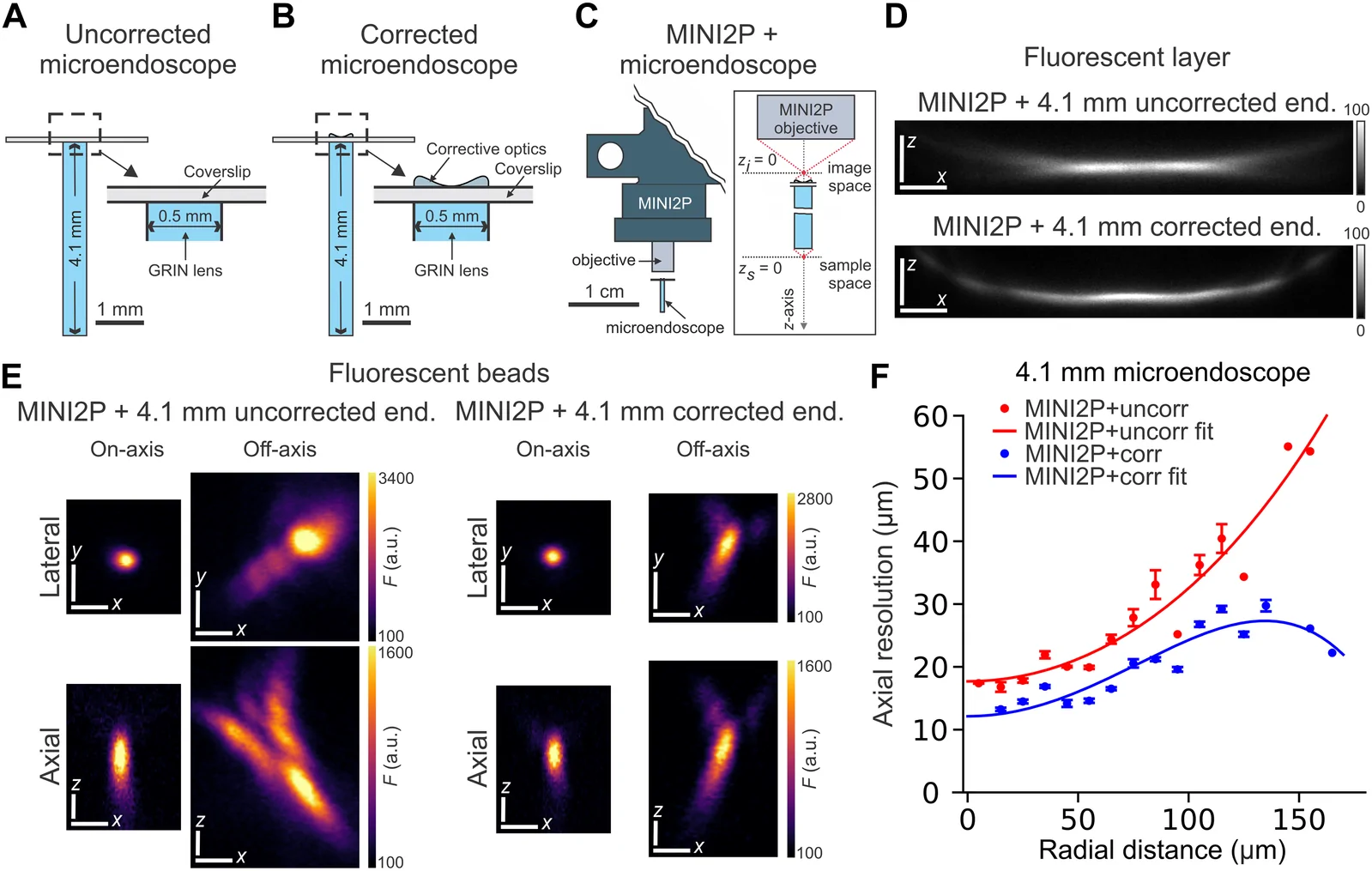
MINI2P coupled with 4.1 mm-long aberration corrected GRIN microendoscopes shows higher and more homogeneous resolution across the FOV. Uncorrected (**A**) or corrected (**B**) microendoscopes based on the 4.1 mm-long GRIN lens. (**C**) Left: optical coupling of MINI2P with GRIN microendoscopes. Right inset: schematic displaying the axial position of focal planes in the image space (z_i_) and in the sample space (z_s_) along the optical axis of the system (*z*-axis). *z*_i_ = 0 and *z*_s_ = 0 correspond to nominal focal lengths of corrected microendoscopes. (**D**) Normalized *x* (horizontal axis), *z* (vertical axis) average intensity projection of a *z*-stack acquired through a homogeneous subresolved fluorescent layer using the MINI2P coupled with an uncorrected (top) or a corrected (bottom) microendoscope. Scale bars: 50 pixels. (**E**) Representative *x*,*y* (lateral) and *x*,*z* (axial) average intensity (*F*) projections of *z*-stacks acquired on subresolved fluorescent beads using the MINI2P coupled with an uncorrected (left) or a corrected (right) microendoscope. Left, On-axis, radial distance d = 3.7 μm and d = 16.6 μm for the uncorrected and corrected system, respectively. Right, off-axis, radial distance d = 113.1 μm and d = 114.6 μm for the uncorrected and corrected system, respectively. Scale bars: *x* and *y*, 3 μm; *z*, 10 μm. (**F**) Axial resolution as a function of the radial distance for MINI2P coupled to uncorrected (red, MINI2P + uncorr) or corrected probes (blue, MINI2P + corr). Data are mean values ± SEM from *n* = 3 experiments. Solid lines are quartic functions fitted to data (table S1).

However, the corrected endoscopes that we initially tested were 4.1 mm long and their length does not allow reaching the most ventral regions of the mouse brain, which can lay ≥5 mm down the outer surface of the brain. We therefore extended the optical characterization described above to the MINI2P coupled to uncorrected and corrected 6.4 mm-long GRIN microendoscopes (*29*), which enable imaging the deepest region of the mouse brain. We found that 6.4 mm-long corrected microendoscopes coupled to MINI2P also enlarged the effective imaging FOV and displayed more homogenous spatial resolution across the FOV compared to uncorrected 6.4 mm-long microendoscopes (Fig. 2, A and B, and fig. S3).

**Fig. 2.**
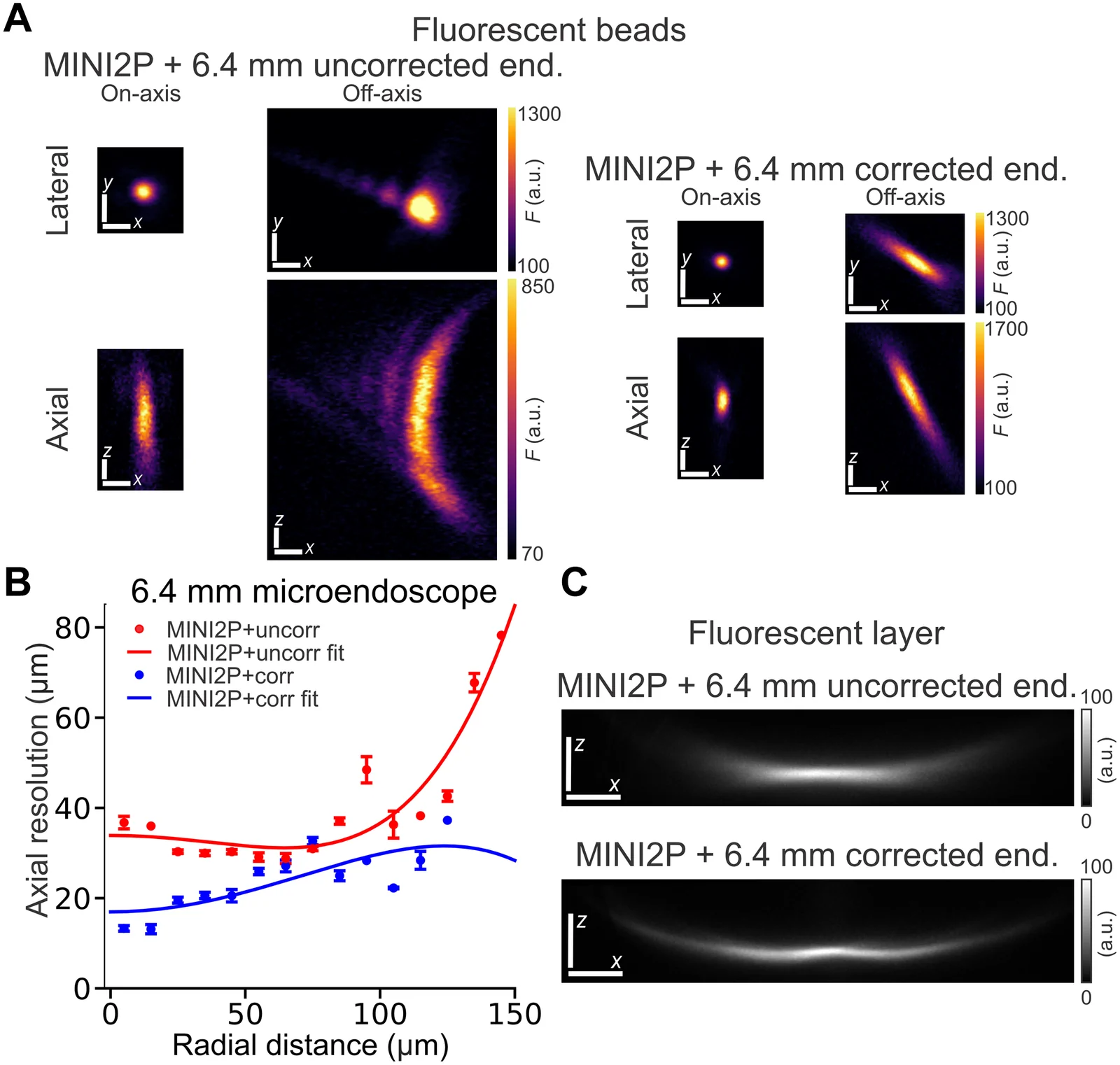
MINI2P coupled with 6.4 mm-long aberration corrected GRIN microendoscopes shows higher and more homogeneous resolution across the FOV. (**A**) Representative *x*,*y* (lateral) and *x*,*z* (axial) average intensity (*F*) projections of *z*-stacks acquired on subresolved fluorescent beads using the MINI2P coupled with an uncorrected (left) or a corrected (right) microendoscope. Left, on-axis, radial distance d = 0.4 μm and d = 4.7 μm for uncorrected and corrected system, respectively. Right, off-axis, radial distance d = 130.5 μm and d = 121.1 μm for uncorrected and corrected system, respectively. Scale bars: *x* and *y*, 3 μm; *z*, 10 μm. (**B**) Axial resolution as a function of the radial distance for MINI2P coupled to uncorrected (red, MINI2P + uncorr) or corrected probes (blue, MINI2P + corr). Data are mean values ± SEM from *n* = 3 experiments. Solid lines are quartic functions fitted to data (table S1). (**C**) Normalized *x* (horizontal axis), *z* (vertical axis) average intensity projection of a *z*-stack acquired through a homogeneous subresolved fluorescent layer using the MINI2P coupled with an uncorrected (top) or a corrected (bottom) microendoscope. Scale bars: 50 pixels.

We then asked whether the enlargement of the imaging FOV extended beyond the nominal focal plane of the GRIN microendoscope to adjacent axial planes. To address this question, we acquired *z*-stacks across a subresolved homogeneous fluorescent layer centered at different axial positions relative to the GRIN nominal focal plane using the tunable *z*-focusing system of the MINI2P (see Materials and Methods). The measurements were performed with the MINI2P coupled to either corrected or uncorrected microendoscopes (fig. S4A). When using uncorrected probes, we observed that the transversal profiles of the fluorescent layer acquired at different axial distances from the focal plane of the GRIN microendoscope were bright and clearly distinguishable from each other only in the central portion of the FOV, while they became progressively blurred and overlapped towards the lateral portions of the FOV. In contrast, the MINI2P coupled to corrected microendoscopes produced well-separated profiles with more uniform thickness and intensity across an enlarged FOV over an axial range of approximately 60 μm in the sample space (fig. S4). These results demonstrate that aberration correction also benefits volumetric imaging with the MINI2P by preserving high axial resolution and an enlarged usable FOV over a depth range corresponding to approximately four neuronal cell layers.

We next investigated how the improved optical properties of the MINI2P coupled to the corrected microendoscopes that we described above translated into better ability to image brain cells (Fig. 3). Towards this goal, we first used the uncorrected and the corrected systems to image neurons expressing a fluorophore in fixed brain slices. When using the uncorrected system, we found strongly aberrated or hardly detectable structures in the marginal portions of the FOV (Fig. 3, left). In contrast, the neuronal structures that were blurred or not visible in the uncorrected system were resolved up to the most marginal portions of the FOV when using the corrected system (Fig. 3, right), a result in agreement with the enlarged effective FOV and more homogeneous axial resolution identified in our initial optical characterization.

**Fig. 3.**
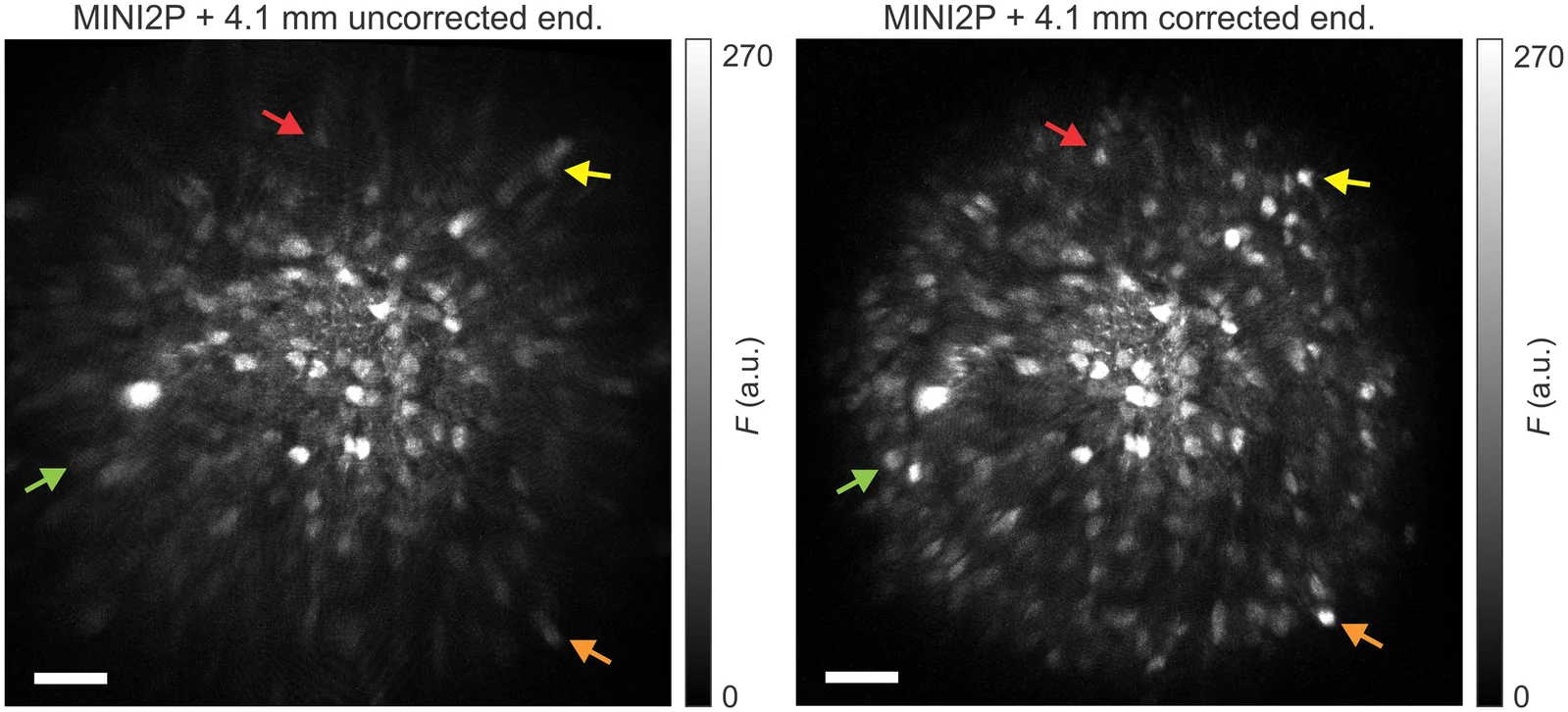
Extended FOV MINI2P imaging using corrected GRIN microendoscopes. Maximum fluorescence intensity (*F*) projections of *z*-stacks of jGCaMP7f-stained neurons in a brain slice, acquired using MINI2P coupled to a 4.1 mm-long uncorrected (left) or corrected (right) microendoscope. Scale bars: 50 μm. Color-matched arrows indicate the same neuron under the two experimental conditions.

### Extended FOV 2P deep brain imaging in freely moving animals

We validated the coupling of the MINI2P with corrected microendoscopes for deep brain imaging in freely moving mice. We expressed the calcium indicator jGCaMP7f in excitatory neurons of the VPM, a sensory thalamic nucleus laying >3 mm down the surface of the brain (Fig. 4, A and B) and coupled the MINI2P with the 4.1 mm-long corrected GRIN microendoscope. We recorded neuronal activity during 15 minute-long open field navigation sessions in a square arena (Fig. 4C, movies S1 and S2).

**Fig. 4.**
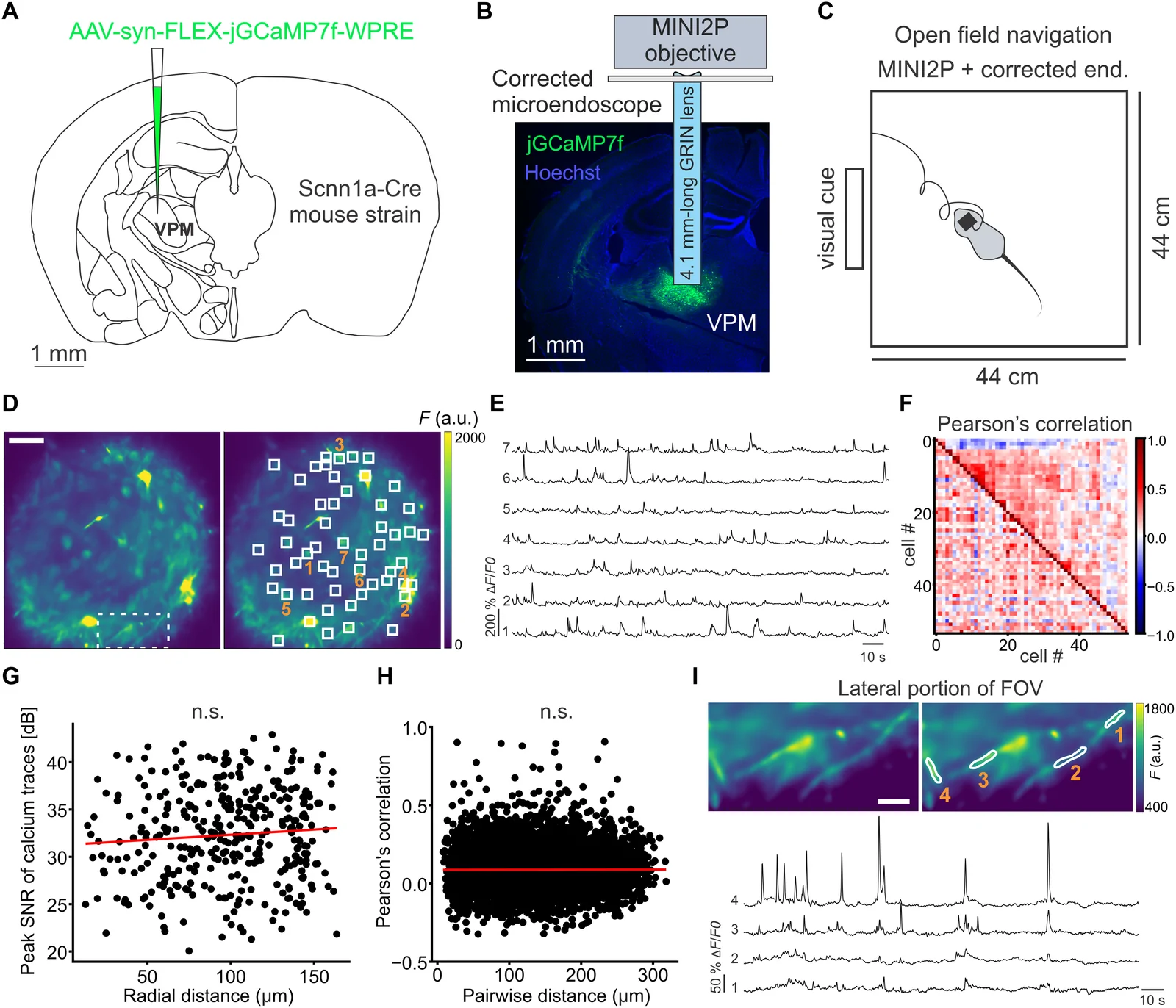
Extended FOV imaging of thalamic activity during open field navigation. (**A**) Schematics of the viral strategy for expression of jGCaPM7f in the VPM. (**B**) jGCaMP7f expression in VPM neurons (green) and corresponding position of the GRIN microendoscope. Cell nuclei were stained with Hoechst (blue). (**C**) Schematics of MINI2P + corrected microendoscope imaging in mice during open field navigation. (**D**) Left: representative FOV of denoised data (see Materials and Methods). Scale bar: 50 μm. Right: detected cell identities are surrounded by white rectangles (*n* = 54 cells). (**E**) Fluorescence traces for cellular identities marked with ordinal numbers in (D). (**F**) Lower-left triangle: Pearson’s correlation matrix for detected cells in (D). Upper-right triangle: corresponding hierarchical clustering. (**G**) Peak SNR of fluorescence traces over radial distance (*n* = 353 cells from *m* = 8 FOVs). Red line: linear regression. Slope being different from 0 was tested with permutation test; n.s., not significant, *P* value = 0.14. (**H**) Pearson’s correlation between pairs of neurons as a function of intercellular distance (*n* = 8581 pairs from *m* = 8 FOVs). Red line: linear regression. Slope being different from 0 was tested with a permutation test; *P* value = 0.79. (**I**) Top left: zoom-in view of the portion of the FOV indicated in (D) with the white dashed rectangle. Scale bar: 20 μm. Top right: white solid contours indicate segmented portions of neuronal processes. Bottom: calcium signals extracted from segmented neuronal processes.

Coupling the MINI2P to corrected microendoscopes enabled imaging cells homogeneously distributed across the FOV (Fig. 4, D to F). The peak SNR of fluorescence traces extracted from both raw and denoised (*33*) movies was independent on the radial distance (fig. S5D and Fig. 4G and for raw and denoised, respectively), indicating homogeneous sampling of neuronal activity across the population, irrespective of cell location in the FOV. The Pearson’s correlation between the fluorescence traces of pairs of neurons was independent on the distance between the neurons of the pair (Fig. 4H and fig. S5E). Furthermore, using the corrected system we were able to visualize neuronal activity in cellular processes located in the most lateral portions of the FOV (Fig. 4I and fig. S5F). All these observations were in agreement with the finding that aberration correction in GRIN microendoscopes increased spatial resolution, especially off-axis (Figs. 1 and 2), leading to homogeneous SNR and preventing cross-talk between adjacent emitters, thus avoiding biases in the computation of correlated neuronal activity (*28*, *29*).

### Spatial mapping of HD tuning in the sensory thalamus

We finally used the unique optical properties of the corrected MINI2P system to reveal the spatial organization of HD coding in the VPM (Fig. 5A) during open field navigation (fig. S6). Recent electrophysiological evidence showed HD tuning in a fraction of VPM cells (*30*). Using the corrected optical system, we confirmed that a small subpopulation of VPM cells was significantly tuned for HD (HD cells, ∼8%, Fig. 5, B and C and fig. S7, see Materials and Methods).

**Fig. 5.**
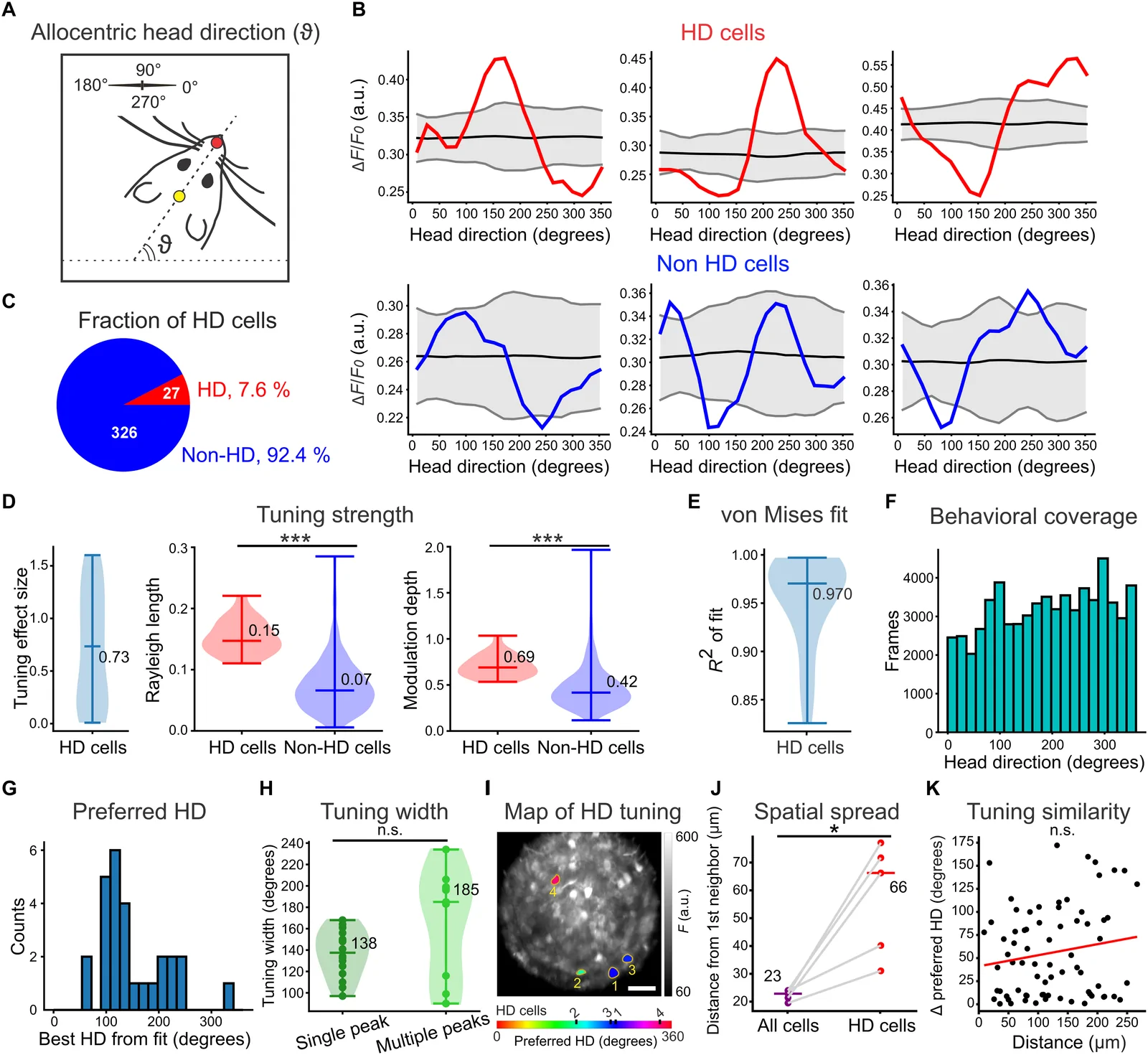
Spatial mapping of HD tuning in the VPM during open field navigation revealed by using the MINI2P coupled to corrected microendoscopes. (**A**) Definition of allocentric HD. Yellow and red spots mark mouse head midpoint and nose, respectively. (**B**) Tuning curves for representative VPM neurons tuned (red, HD cells) or not tuned (blue, Non-HD cells) to HD. Black curves and gray-shaded areas represent the average and SD of shuffled tuning curves. (**C**) Fraction of HD cells over all imaged VPM neurons (*n* = 353, *m* = 8 FOVs from 6 mice) based on Rayleigh length criterion. (**D**) Left: distribution of effect size of tuning strength for HD cells. Center: distribution of Rayleigh length values for HD (red) and non-HD cells (blue). *P* < 10^−15^, Mann-Whitney U test. Right: distribution of modulation depth values for HD (red) and non-HD cells (blue). *P* < 10^−9^, Mann-Whitney U test. (**E**) Distribution of R^2^ values of von Mises models fitted to HD tuning curves. (**F**) Distribution of behavioral HD angles across all data. (**G**) Distribution of preferred HD for HD cells. Median: 128°. (**H**) Tuning width for HD cells with a single (*n* = 16) or multiple peaks (*n* = 11). *P* = 0.13, Mann-Whitney U test. (**I**) Average intensity (*F*) projection of a representative FOV (4 HD cells/51 cells). HD cells are color-coded based on preferred HD. Scale bar: 50 μm. (**J**) Median distance between one cell and its first neighbor across all cells and HD cells for each FOV (*n* = 5 FOVs with at least 2 HD cells per FOV). *P* = 0.035, paired *t* test. (**K**) Angular difference between preferred HD of HD tuned cell pairs as a function of intercellular distance (*n* = 73 pairs from *m* = 8 FOVs). Red line: linear regression. *P* = 0.16, permutation test.

Tuning strength of HD cell population was broadly distributed (Fig. 5, D to E) and, despite the fact that during navigation mice covered the whole 0°-360° HD range at the behavioral level (Fig. 5F), preferred HDs appeared unevenly distributed in the imaged FOVs (Fig. 5G). Tuning to HD was quite broad (Fig. 5H) as reported in previous electrophysiological characterization (*30*).

Importantly, leveraging on the unbiased structure-function correlation that the corrected MINI2P system enabled, we visualized the spatial organization of HD tuned neurons in the VPM (Fig. 5I). To test whether HD cells were clustered in space, we measured the distance between each cell and its nearest neighbor, either considering only the subpopulation of HD tuned neurons or considering the whole population of imaged neurons, as a control. Across FOVs, we found that the median distance between one cell and its first neighbor was significantly higher within the subpopulation of HD cells compared to the whole neuronal population (spatial spread, Fig. 5J), indicating the absence of spatial clustering of the HD cell population. We also found no dependence of the relative angular difference between the preferred HD of pairs of HD cells on their intercellular distance (tuning similarity, Fig. 5K), suggesting the absence of evident spatial clustering of HD preference at least within the imaged FOVs. Similar results were obtained using the modulation depth (see Materials and Methods) as metric for determining HD tuning (fig. S7). Analysis for HD tuning on raw data (fig. S8) confirmed the broad tuning for HD (fig. S8, E and M) and significant difference in distance between one cell and its first neighbor for HD cells compared to all cells within the FOV (fig. S8, F and N).

## DISCUSSION

The ability to record activity from large neuronal populations with high spatial resolution during natural behavior is essential to understand how distributed circuits support complex brain functions (*1*–*3*). MINI2Ps have emerged as powerful tools for this purpose, enabling cellular-resolution imaging in freely moving animals (*19*–*22*, *34*–*38*). However, when used to image deep brain regions, MINI2Ps typically rely on GRIN-based microendoscopes (*21*), which introduce substantial optical aberrations that limit spatial resolution and restrict the FOV (*28*, *29*). In this study, we addressed these limitations by integrating lightweight microfabricated corrective optics into GRIN microendoscopes, demonstrating that aberration correction markedly improves imaging performance and expands the accessible FOV during deep brain MINI2P imaging.

Our optical characterization experiments revealed that uncorrected GRIN microendoscopes, when coupled to the MINI2P, produced pronounced off-axis distortions, resulting in a rapid degradation of axial resolution with increasing radial distance. In contrast, our corrected microendoscopes, when coupled to the MINI2P, exhibited a more uniform axial resolution across an enlarged FOV and eliminated many off-axis distortions. Importantly, these improvements translated into a more than fourfold increase in effective FOV, allowing higher resolution imaging even at the periphery of the FOV. Previous work (*28*, *29*) has demonstrated that optical aberrations have direct consequences for the quality and interpretability of functional imaging data. For example, the improved and more homogenous spatial resolution across the FOV of corrected GRIN microendoscopes coupled to benchtop 2P microscopes resulted in increased SNR and reduced cross-contamination between neighboring emitters (*28*, *29*). These factors are critical when quantifying neuronal population dynamics, particularly for measures such as pairwise correlations (*28*, *29*), which are highly sensitive to optical blur and signal mixing. Previous work and the findings reported herein, therefore, highlight that correcting GRIN-induced aberrations is not merely a technical refinement, but a necessary step to achieve accurate and unbiased measurements of neural activity in deep brain regions in both benchtop and MINI2Ps.

Interestingly, coupling corrected microendoscopes to the MINI2P improved axial resolution compared to the MINI2P coupled to uncorrected microendoscopes not only off-axis, but also on-axis. This behavior differed from previous studies using conventional benchtop 2P microscopes, in which aberration correction primarily improved off-axis image quality while leaving the on-axis resolution largely unchanged (*28*, *29*). Additionally, the on-axis spatial resolution of the MINI2P coupled to the 4.1 mm-long corrected microendoscope was better than that of the MINI2P not coupled to GRIN microendoscopes (table S1). A plausible explanation is that the corrective micro-optics compensated not only for the intrinsic aberrations of the GRIN lens but also, at least partially, for aberrations introduced by the MINI2P optical system. This interpretation is supported by the observation that the measured spatial resolution of the MINI2P equipped with the Domilight D0254 micro-objective (NA = 0.5, lateral resolution ∼1.2–1.5 μm; axial resolution ∼15–16 μm, see (*21*) and this paper) is lower than the theoretical diffraction-limited performance expected from a 0.5 NA objective at 920 nm excitation [lateral resolution ∼0.7 μm; axial resolution ∼6–7 μm (*17*, *39*)]. These findings suggest that aberration-corrected GRIN microendoscopes may partially compensate for imperfections of the miniature microscope itself, extending their benefits beyond correction of GRIN lens aberrations alone.

Several approaches have been proposed to mitigate optical aberrations in GRIN endoscopes imaging, including low-order adaptive optics (*24*), adaptive optics through pupil segmentation (*25*, *26*), and geometric transformation adaptive optics (*27*). However, all the above mentioned methods require substantial modification of the optical set-up and/or the development of specific software control (*24*–*27*). Alternatively, a correction objective lens was specifically designed to compensate for the aberrations of a 0.5 mm-diameter, 6.7 mm-long GRIN lens using benchtop 2P microscopes (*40*), but corrected miniaturized objectives for GRIN lens-based 2P imaging in freely moving experiments are currently not available. Recent advances in microfabrication techniques, particularly 2P lithography (*31*), enabled the design and integration of custom optical elements at the microscale (*41*). These technologies provide an opportunity to engineer miniaturized corrective optics that can be directly incorporated into GRIN-based systems (*28*, *29*), enabling precise compensation of aberrations without substantially increasing system dimensions (corrective optical element volume < 0.5x0.5x0.07 mm^3^) and weight (corrective optical element weight < 0.5 mg). Therefore, such miniaturized optics can be implemented without major modifications to existing imaging architectures and is ideally designed to be compatible with miniaturized microscopes, making them attractive for widespread adoption.

Although aberration corrected GRIN microendoscopes substantially improve the optical performance of deep imaging in freely moving animals, our results also potentially highlight a limitation of some of the current portable 2P imaging systems. In fact, we found no combination of MINI2P and thin GRIN microendoscopes that enabled imaging beyond approximately 5 mm from the brain surface while maintaining the same spatial resolution achieved in more superficial regions (see on-axis spatial resolution of the MINI2P coupled to corrected 4.1 mm-long microendoscopes and on-axis spatial resolution of the MINI2P coupled to corrected 6.4 mm-long microendoscopes). Our findings suggest that this limitation no longer arises primarily from the GRIN microendoscope itself, but rather from the optical performance of the miniature microscope. In particular, the measured spatial resolution of the MINI2P used in this study (*21*) is currently lower than the theoretical diffraction-limited performance expected for an optical system with a nominal numerical aperture of ∼0.5 (*17*, *39*). This interpretation is further supported by our previous work, which demonstrated that the aberration corrected GRIN microendoscopes used in this study achieved axial resolutions below 10 μm when coupled to conventional benchtop 2P microscopes (*28*, *29*). Together, these observations indicate that further improvements in deep brain imaging in freely moving animals will likely require the development of next-generation miniature objectives, or a redesign of the optical train of portable 2P microscopes, to increase the effective numerical aperture and reduce intrinsic system aberrations. Such advances, combined with aberration corrected GRIN microendoscopes, would enable high-resolution optical interrogation of increasingly deeper brain regions while preserving the minimal invasiveness afforded by thin microendoscopic probes.

We validated the performance of the corrected system in vivo by imaging neuronal activity in the VPM of the thalamus in freely moving mice. This region lies more than 3 mm below the brain surface and has been largely inaccessible to cellular-resolution 2P imaging during natural behavior. The expanded FOV of our method enabled the simultaneous recording of large numbers of neurons, despite the fact that the diameter of the implanted microendoscope was limited to 500 μm. Hence, corrected microendoscopes provide a comprehensive view of neural population activity while mitigating the damage to the brain tissue introduced by the implantation of larger cross section GRIN lenses or other larger optical elements that have been previously used in combination with MINI2Ps to reach deep brain structures in behaving animals (table S4). Moreover, the observation that SNR and correlation measures were independent of radial position within the FOV indicated that the corrected system enabled uniform sampling of neuronal populations, thereby reducing spatial biases that may confound the interpretation of network activity. Importantly, our optical approach allowed the investigation of the spatial organization of HD coding in the thalamus, revealing that HD-selective neurons are sparsely distributed and do not exhibit clear spatial clustering within the imaged region. While previous electrophysiological studies identified HD tuning in subsets of neurons in the VPM (*30*), our approach provides cellular-resolution mapping of these signals, demonstrating the power of large-scale and high-resolution optical recordings for uncovering circuit organization.

Finally, the ability to image neuronal processes in the most peripheral regions of the FOV further underscores the advantages of aberration correction in GRIN microendoscopes, as demonstrated in benchtop 2P microscopes (*42*). Fine structures are particularly susceptible to optical distortions, and their reliable detection is essential for studying the dynamics of subcellular activity and morphology. The improved resolution achieved with corrected microendoscopes opens new opportunities to investigate how neuronal signals propagate across dendritic and axonal compartments in deep brain circuits, a question that has remained largely unexplored in freely behaving animals.

In summary, we used 3D printed micro-optics to address the fundamental limitations introduced by aberrations of GRIN microendoscopes during MINI2P deep brain imaging in freely moving mice. We demonstrated up to more than fourfold increase in FOV and we used the MINI2P coupled to corrected microendoscopes to describe the spatial organization of HD tuning in the mouse sensory thalamus. This method is ideally designed for freely moving animal preparations as it is lightweight, has small footprint, and is built-in in the GRIN microendoscope, requiring no major modification of MINI2P architecture. Coupling MINI2P with aberration corrected GRIN-based microendoscopes is therefore a powerful and ready-to-use method that will enable neuroscientists to explore new questions regarding high-resolution mapping of population activity over enlarged FOVs in deep brain regions of freely moving animals.

## MATERIALS AND METHODS

### Assembly of MINI2P

MINI2P was assembled as described in (*21*), using the following optical components: a quartet μTlens (Silver IRMS-PIF.15.P02, poLight, Tønsberg, Norway), a MEMS scanner (MEMS-L A3I12.2-1200AL, Mirrorcle Technologies Inc., Richmond, CA, U.S.), a scan lens (D0166, Domilight, Nanjing, China), 3x miniaturized objectives with 0.45–0.5 NA (D0254 or D0309, Domilight), and a dichroic mirror (DMSP0405, Sunlight Technology Co., Fuzhou, China). The excitation fiber (HC-920 p.m., NKT Photonics, Copenhagen, Denmark; fiber length, 2.2 m) was coupled with the following 920 nm pulsed laser sources (80 MHz repetition rate): Alcor 920–1 (Spark Lasers, Martillac, France) or Axon 920–1 TPC (Coherent Corp., Saxonburg, PA, U.S.). Power modulation of Alcor 920–1 was performed with a Pockel cell (model 350-80LA KD*P modulator, Conoptics Inc., Danbury, CT, U.S.), while Axon 920–1 was equipped with an internal total power control module. Before fiber coupling, positive group delay dispersion (GDD) was introduced in the laser path by adding one or three 15 cm-long glass rods (H-ZF62, Sunlight Technology Co.) for Axon 920–1 TPC or Alcor 920–1, respectively. For both laser sources, optimization of the pulse duration at the fiber output was performed using the laser internal module for GDD compensation. Fluorescence signals emitted by the sample and collected by the objective passed through a tapered fiber bundle (Mini2PCollection Fiber v1, LABmaker, Berlin, Germany), a short pass filter (FESH750, Thorlabs, Newton, NJ, U.S.), a dichroic mirror (DMLP567R, Thorlabs), and an emission filter (MF525–39, Thorlabs) before being focused on the active area of a GaAsP photomultiplier tube (PMT2101/M, Thorlabs).

### Assembly of GRIN microendoscopes

Two types of commercially available customized GRIN rods with 0.5 NA, 500 μm diameter and lengths 4.1 mm and 6.4 mm (NEM-050-43-00-810-S-1.0p and NEM-050-25-10-860-S-1.5p, GRINTECH GmbH, Jena, Germany, respectively) were glued perpendicularly to the bottom surface of a round glass coverslip (diameter, 5 mm or 3 mm; thickness, 200 μm or 100 μm for 4.1 mm- or 6.4 mm-long GRIN rods, respectively) using UV-curable glue (NOA63, Norland Products, Cranbury, NJ, U.S.). Corrected probes differed from uncorrected probes only for the presence of the corrective optical element. The prototype corrective optical element was initially fabricated with 2P lithography and then replicated with NOA63 on the top surface of the glass coverslip using a molding procedure (see (*28*, *29*) for design of the corrective optical element and technical details). For optical characterizations, 5 mm-diameter coverslips were used and secured to custom metallic rings (7 mm external diameter) to ease handling the probe, while for in vivo measurements 3 mm-diameter coverslips were attached to 3D printed plastic rings (4.5 mm external diameter) compatible with a commercial GRIN probe holder (Proview holder, Inscopix Inc. Mountain View, CA, U.S.) to facilitate stereotaxic implantation in the mouse brain.

### Optical performance of MINI2P coupled to microendoscopes

For optical characterizations, MINI2P equipped with the 3x air objective D0254 was coupled with uncorrected or corrected microendoscopes using a custom optomechanical set-up designed for precise alignment of the GRIN lens-based probe with respect to the optical axis of the MINI2P objective and for fine adjustment of the distance between the objective lens and the upper surface of the microendoscope. The set-up was composed of a 3D printed custom holder mounted on a dual-axis manual goniometer (part no. GNL20/M, Thorlabs Inc., Newton, NJ, U.S.) and a XYZ micrometric manual linear stage (part no. MS3/M, Thorlabs). Unless otherwise stated, the focal distance in the image space was set to 100 μm for uncorrected probes and to 150 μm for corrected probes (nominal focal distance) and the focal plane was never moved using the fast *z*-focusing system of the MINI2P. The whole system was mounted on a motorized micrometric linear stage (linear stage, part no. PT1/M, equipped with a motorized actuator, part no. Z925D, Thorlabs) to move both the MINI2P and the microendoscope along the vertical direction, while their reciprocal distance remained fixed, and perform *z*-stacks of calibration samples. The motorized actuator was directly integrated and driven by ScanImage (MBF Bioscience, Williston, VT, U.S.), the software used to control MINI2P scanning and acquisition. The medium between the MINI2P objective and the microendoscope was air, whereas the one between the front end of the GRIN rod and the sample was distilled water for subresolved fluorescent layers, calibration grid, and subresolved fluorescent beads, or phosphate-buffered saline (PBS) for mouse fixed brain slices. Due to the short working distance of GRIN lenses, no coverslips were placed over the samples, rather a thin layer of optical glue (NOA63) was deposited and solidified to keep layers and beads firmly in place during image acquisition.

#### Subresolved fluorescent layers

Subresolved fluorescent layers (thickness, ∼1 μm) were obtained as described in (*28*) and mounted on a microscope slide. We acquired *z*-stacks at 512 pixels by 512 pixels resolution with 1x digital zoom, 2 μm *z*-step, 50 frame averaging, and laser power measured under the objective set to ∼25 mW, ∼40 mW or ∼60 mW for MINI2P alone, MINI2P coupled with the 4.1 mm-long or with the 6.4 mm-long microendoscopes, respectively. For fig. S4, the focal distance in the image space was changed from 30 μm to 210 μm using the μTlens in steps of 20/30 μm and a *z*-stack was acquired through the layer in each condition. We first performed all the *z*-stacks using the MINI2P coupled with a corrected microendoscope. Then, we removed the corrective microlens from the microendoscope coverslip and we repeated the same measurements using the uncorrected microendoscope. *z*-stacks of fluorescent layers were not corrected for FOV distortion (see paragraph “Calibration grid” below). Layer profiles were obtained using the *Reslice* plugin in ImageJ/Fiji applied to a rectangular central portion of the original *z*-stack (widths in *x* and *y* directions: 512 pixels and 15 pixels, respectively) to get a transverse view of the central portion of the *z*-stack and then computing the average along the y direction to obtain the *x*,*z* intensity projection.

#### Calibration grid

To correct MINI2P acquisitions for FOV distortion, we acquired images of a calibration grid with 10 μm spacing (Part no. R1L3S3PR, Thorlabs). To enhance fluorescence signal collection, we deposited on the calibration grid a thin layer of highlighter ink covered by a thin layer of optical glue (NOA63) on the grid. *z*-stacks were acquired using MINI2P alone or MINI2P coupled to a GRIN microendoscope at different pixel resolutions (2048 by 2048, 1024 by 1024 or 512 by 512), with 1× or 2× digital zoom, 2 μm *z*-step, 30 frames averaging and power under the objective set to 2–5 mW. For *z*-stacks performed at digital zoom 1, laser power was exponentially increased from the first plane (center of FOV) to the last plane (edge of FOV), up to ∼10 mW, to counteract the loss in fluorescence signal collection at high radial distances. FOV distortion was measured on maximum intensity projections of grid acquisitions using the *Distortion Detection* ScanImage plugin (*21*). After application of the *Distortion Correction* plugin, which performs a warping transformation on raw data based on previously measured FOV distortion, we estimated the real pixel size from corrected grid images acquired under different imaging conditions (e.g., MINI2P objective type, digital zoom value, and microendoscope type, see table S3 for details). Finally, to correct for FOV distortion the warping transformations were then applied to images of fluorescent beads and brain slices acquired under the same imaging conditions (table S3).

#### Subresolved fluorescent beads

Subresolved fluorescent beads (diameter 1 μm, Fluoresbrite YG Carboxylate Microspheres 1.00 μm, Product no. 15702, Polysciences Inc., Warrington, PA, U.S.) were monodispersed in water (dilution, 1:10^6^) and prepared on a microscope slide pre-treated with poly-l-lysine (0.1 mg/mL, Cat. No. P2636, Merck, Darmstadt, Germany). Fluorescent beads were spread over the FOV area at different radial distances from the optical axis. For each MINI2P coupled with a GRIN microendoscope type (i.e., 4.1 mm-long and 6.4 mm-long), bead measurements were repeated using three different corrected and uncorrected microendoscopes (*n* = 3 independent experiments). For each experiment, we acquired multiple *z*-stacks of the same FOV at 2048 by 2048 pixel resolution with 1× or 2× digital zoom, 1 μm *z*-step, and 30 frame averaging. For each *z*-stack, we varied the laser power measured under the objective in order to obtain the same level of collected fluorescence signal (bead peak intensity ∼3500 a.u, 32 bit signed integer digitization range) from beads located at different radial distances from the center of the FOV (radial distance ranges: central beads, 0–40 μm; pericentral beads, 40–70 μm; lateral beads, 70–100 μm; marginal beads, >100 μm). We adopted this procedure to counteract the progressive signal intensity loss typical of GRIN lenses in the lateral portions of the FOV and ensure a fair comparison of the size of imaged beads, independently of their location within the FOV. The ranges of power values used for 4.1 mm- or 6.4 mm-long microendoscopes, respectively, were: central beads, (13 ± 4) mW or (21 ± 9) mW; pericentral beads, (24 ± 14) mW or (26 ± 6) mW; lateral beads, (34 ± 14) mW or (59 ± 11) mW; marginal beads, (68 ± 8) mW or (68 ± 9) mW (power values were measured in front of the MINI2P objective). To measure the spatial resolution of the MINI2P alone, a single *z*-stack was acquired at 2048 by 2048 pixel resolution, 2× digital zoom, 1 μm *z*-step, 30 frame averaging and power ∼10 mW.

Quantification of PSF size was performed on distortion corrected data using custom code written in ImageJ/Fiji and Python 3.7. First, we computed the *x*,*y* average intensity projection of the *z*-stack in ImageJ/Fiji and we used it to: (i) manually select rectangular region of interest (ROIs) comprising single beads; (ii) determine minimum, maximum, and mean intensity values within each ROI; (iii) extract the coordinates of the center of mass of the bead in pixels to later access its distance from the central pixel of the image. Then, we used a custom ImageJ/Fiji script that carried out the following functions for each ROI: (i) compute the *x*,*y*, *x*,*z*, and *y*,*z* average intensity projections of the ROI; (ii) convert the projections into a binary mask and detect the bead profile; (iii) generate a line along the direction of the principal axis of the bead passing through the center of mass for *x*,*y*, *x*,*z*, and *y*,*z* projections, plus an additional line perpendicular to the first one and passing through the center of mass for the *x*,*y* projection; (iv) perform Gaussian fits of bead intensity profiles along the drawn directions and compute the full width at half maximum (FWHM) of fitted curves. Next, we used custom Python code to: (i) filter the beads suitable for further analyses if the maximum intensity value of the *x*,*y* average projection of the ROI was between 1500–5500 a.u., all four Gaussian fits had R^2^ > 0.9, and were not rejected following visual inspection; (ii) estimate lateral and axial sizes of each selected bead as mean FWHM value between the two values obtained from *x*,*z*, and *y*,*z* bead projections for the axial direction and from *x*,*y* projection along the two axes for the lateral direction; (iii) compute the radial distance of the bead from the center of the FOV in microns. Finally, results from experimental replicates were pooled together, binned based on radial distances from the center of the FOV (bin width: 10 μm), averaged, and fitted with a quartic function *f(x) = ax^4^ + bx^2^ + c* (see table S1).

#### Mouse fixed brain slices

Brain slices were prepared as described below in “Histology” except for the mounting procedure. On the day of the experiment, freshly washed fixed slices (10 minutes in PBS for 3 times) were gently placed over a microscope slide and kept wet with PBS throughout the imaging session. For each experiment, we identified a FOV containing fluorescently tagged neurons (in correspondence with VPM, see below) and acquired a *z*-stack using the MINI2P coupled to a corrected or uncorrected microendoscope at 1024 pixels by 1024 pixels resolution, 1× digital zoom, 5 μm *z*-step, and 8 frame averaging (number of axial steps: 29). The same FOV was imaged using corrected and uncorrected microendoscopes. Acquired *z*-stacks were corrected for FOV distortion and maximum intensity projections were computed in ImageJ/Fiji.

#### Estimation of FOV enlargement

We defined the “effective FOV” radius with two criteria. First, as the radial distance at which the axial spatial resolution became equal to 20 μm (threshold = 20 μm in the second row of table S1). Second, as the distance from the optical axis at which the axial resolution of the system was equal to the on-axis axial resolution of the uncorrected system +10% (third row of table S2), determined using the quadratic functions fitted to axial resolution data (table S1). The enlargement of FOV was computed as ratio between the radii of the effective FOV of the corrected and uncorrected system calculated with the second criterion.

### Animals

All experimental procedures performed on animals were approved by the National Council on Animal Care of the Italian Ministry of Health (authorization # 630/2023-PR and # 826/2024-PR) and were carried out according to the guidelines of the Animal Health Regulatory Committee of the Istituto Italiano di Tecnologia, to the National legislation (D.Lgs. 26/2014), and to the legislation of the European Communities Council Directive (European Directive 2010/63/EU). The B6;C3-Tg(Scnn1a-cre)3Aibs/J mouse line (here called Scnn1a-Cre) was purchased from The Jackson Laboratory (strain # 009613, Bar Harbor, ME, U.S.) and bred with C57BL/6 mice. Animals were housed with unrestricted access to water and food under a 12-hour light-dark cycle. At postnatal day 25, animals were removed from the original cage and housed in groups of up to five subjects of the same sex. Transgenic mice (males) were used for experiments.

### Viral injection and microendoscope implantation

Surgical procedures were performed on Scnn1a-Cre mice starting from 14 weeks of age. For viral injection in the VPM, mice were anesthetized, placed onto a stereotaxic apparatus (Stoelting Co., Wood Dale, IL, U.S.), and maintained anesthetized under 1.5% isoflurane in 0.9 L/min oxygen at 37°C. After scalp incision, a small craniotomy was drilled at the following coordinates with respect to bregma: AP, −1.6 mm; ML, +1.8 mm. A glass micropipette containing the viral solution (pGP-AAV-syn-FLEX-jGCaMP7f-WPRE, a gift from Douglas Kim & GE, NIE Project, Addgene viral prep # 104492-AAV1, Addgene, Watertown, MA, U.S.; dilution, 1:4 in saline) was slowly (∼0.5 mm/min) lowered to a depth of 3.3 mm from the brain surface and a volume of 0.8 μl was injected at 50 nL/min using an hydraulic pump (UltraMicroPump UMP3, World Precision Instruments, Sarasota, FL, U.S.). Five minutes after the end of the injection, the pipette was slowly retracted and the head skin sutured and treated with Iruxol (Smith & Nephew GmbH, Hamburg, DE). Starting from 10 days after the viral injection, mice underwent stereotaxic implantation of the 4.1 mm-long corrected microendoscope assembled with the 3D printed plastic ring (see paragraph “Assembly of GRIN lens-based microendoscopes” above). Before surgery, a bolus of Dexamethasone (dilution, 1:5 in saline; volume, 100 μL/10 g mouse weight, Dexadreson, MSD Animal Health, Rahway, NJ, U.S.) was administered via intramuscular injection in the hind limbs. A craniotomy of about 600 by 600 μm^2^ was performed at the following coordinates relative to bregma: AP, −1.6 mm; ML, +1.7 mm. To generate a track for microendoscope insertion, a blunt needle with the same diameter of the used GRIN lens (0.5 mm) was slowly inserted into the tissue to a depth of 2.9 mm from the brain surface, while keeping the insertion site wet with saline. After 5 min, the needle was slowly retracted. The same procedure was repeated a second time replacing the blunt needle with the corrected GRIN microendoscope. The GRIN microendoscope was fixed to the stereotaxic frame using the commercial Proview holder and lowered to a depth of 3.2 mm from the brain surface. To fix the GRIN microendoscope to the mouse head, first some drops of a non-toxic surgical adhesive (3 M Vetbond, ID 7100139485, 3 M, Saint Paul, MN, U.S.) were gently placed between the bottom surface of the microendoscope coverslip and the skull. The plastic ring of the GRIN microendoscope was then cemented to the skull using dental cement (Super-bond C&B Kit, Sun Medical Co., Moriyama, Japan). Finally, a custom metal head bar was secured to the skull using cyanoacrylate glue (Loctite Super Attack Power Gel, Henkel, Düsseldorf, Germany) and dental cement. At the end of the surgery, mice received an intraperitoneal injection of Baytril (volume, 100 μL; Bayer, Leverkusen, Germany) before returning to the cage and being housed separately.

### Behavioral apparatus for open field navigation

The behavioral apparatus for open navigation sessions was a 44 by 44 cm^2^ plastic box with 34.5 cm high grey walls. The box was positioned 34.5 cm from the ground level and had a transparent Plexiglas floor. A 15 by 15 cm^2^ square of white paper was positioned on one of the walls as a polarizing visual cue for mouse orientation in the arena. A CMOS camera (Basler ace acA800-510um, Basler, Germany) equipped with an 8 mm objective (Computar, Durham, NC, USA) was used to record mouse behavior under the control of Pylon software (version 7.5, Basler, Germany). Environmental illumination was provided by a visible red lamp.

### In vivo MINI2P recordings

Starting from 50 days after AAV injection, mice were habituated to the experimenter and to head fixation while sitting or running on a wheel in daily sessions of increasing duration (from 1 to 40 min in 5–6 days). Next, mice first underwent baseplating [i.e. installation of a mechanical support for the MINI2P scope body on the mouse head, see (*21*)] and then were habituated to navigate in the open arena before starting MINI2P experiments.

#### Baseplating

The animal was head-fixed while having the possibility to run on a wheel. The baseplate was screwed to the MINI2P scope body, which was held by a mechanical support equipped with five degrees of freedom (3-axis translation provided by three linear stages, part no. PT1/M, Thorlabs; tip and tilt rotation provided by dual-axis goniometer, part no. GNL20/M, Thorlabs). The MINI2P equipped with the 3x miniaturized objective D0309 was then finely positioned such that its optical axis was aligned with the optical axis of the GRIN lens. Once the targeted focal plane was identified, the MINI2P was moved 30–50 μm upward along the vertical direction, and the baseplate was cemented to the animal head.

#### Habituation to open field arena

Starting from 3 days after baseplating and for 4–5 consecutive days, the mouse was briefly head-fixed on the wheel and a plastic dummy scope with the same shape and weight of the MINI2P was positioned on the baseplate. The dummy scope was attached to the head fixation set-up via a dummy fiber to mimic experimental tethering conditions. The mouse was then placed inside the behavioral arena for daily sessions. Daily sessions increased in duration over days from 5 to 20 min, until the animal comfortably navigated the space while carrying the dummy scope on its head.

#### Open field navigation

On the day of the experiment, the mouse was briefly head-fixed on the wheel and the MINI2P equipped with the 3x D0309 objective was secured to the baseplate on the animal head. The microendoscope focal plane was identified using the fast *z*-focusing system of the MINI2P (μTlens, range of axial displacement compared to baseplating: 0 μm/−60 μm, 8 FOVs). Laser power was adjusted to obtain a well-populated single-frame histogram at a frame rate of 7.5 Hz (average power range: 80–120 mW). Next, the mouse was gently placed inside the square arena. 2P imaging recordings (bidirectional scan with frame rate 7.5 Hz, 16 bits, 512 by 512 pixel resolution, 1× digital zoom, and no frame averaging) and the camera behavioral imaging started. The trigger for camera recordings (4 acquired frames every trigger, exposure 32,000 μs) was provided by the start signal of every MINI2P frame acquisition to obtain a final frame rate of 30 Hz. Experiments were carried out in a silent dark room, where faint illumination for mouse navigation was provided only by a red lamp (see paragraph “Behavioral apparatus for open field navigation” above). A typical recording session lasted ∼15 min during which the animal freely navigated the arena. At the end of the experiment, the mouse was gently collected from the box, briefly head-fixed again to remove the MINI2P from the baseplate and returned to its home cage.

### Analysis of in vivo MINI2P recordings

#### Tracking of animal behavior

The Social LEAP Estimates Animal Poses (SLEAP – V 1.3.3) (*43*) software was used to track animal position from the behavioral videos (see paragraph “Behavioral apparatus for open field navigation” above). A total of eight anatomical keypoints were tracked with SLEAP: nose, head midpoint, right ear, left ear, neck, body center, mid back end, and tail base. The output of SLEAP included the positions in pixels of these anatomical keypoints for each frame, resulting in eight keypoint time traces. Two SLEAP models were independently trained with at least 400 manually labeled frames from multiple recordings involving different animals and illumination conditions. In frames in which the prediction of one keypoint failed, the location of the missing keypoint was estimated by linear interpolation with the location of the nearest correctly resolved temporal neighbors (*43*). Each keypoint trace (*t*) was then smoothed with a moving average with a temporal window of 0.25 s. The kinematic variables used to describe animal behavior along the recording were computed as follows (with *t_i_* representing *t* in the *i*-th video frame): (i) for instantaneous linear speed $\left(S_{t_{i}}\right)$, first *x*,*y* components were calculated from the *x*,*y* positions of the head midpoint using the Python function *numpy.gradient* and then their L2 norm was computed; (ii) inter-frame running distance was computed as $D_{t_{i}}$ = $S_{t_{i}}\cdot$Δ*t* where Δ*t* = 1/30 Hz; (iii) accumulated distance over time was computed as the cumulative sum of all inter-frame distances within a session; (iv) allocentric HD ϑ was defined for each frame as the anti-clockwise angle formed by the line connecting the head midpoint to the nose of the mouse and the reference line that represented the 0°–180° direction in the square arena (Fig. 5A). ϑ was computed identifying the direction head midpoint ➔ nose using the coordinates of the two keypoints and applying the Python function *numpy.arctan2*. To evaluate the coverage of the behavioral arena during a navigation session, the arena was subdivided into 484 square spatial bins (size, 2 cm by 2 cm). The coverage was computed as the number of visited bins divided by the total bin number, where a bin was considered visited if the animal midpoint belonged to that bin for at least one frame.

#### Preprocessing of MINI2P data

Each raw MINI2P recording was first corrected using the rigid motion correction of CITE-ON (*44*). Corrected recordings were then denoised with a deep self-supervised denoising algorithm (*33*) to decrease shot noise and enhance SNR of collected fluorescence signals. In a fraction of the data, the piecewise rigid motion correction routine of CaImAn (*45*) was also applied. Mean intensity projection of denoised t-series was next computed and used for detection of cell bodies using bounding boxes in CITE-ON (*44*). Extraction of fluorescence traces *F*(*t*) was performed with CITE-ON computing the average fluorescence intensity value between the 80th and the 95th or 100th percentile of the pixel intensity distribution within the box for raw or denoised data, respectively, and subtracting the background value frame-by-frame (*44*). The background level was estimated as the average intensity value between the 10th and the 25th percentile of the pixel intensity distribution within the box (*44*). Using custom Python code, the baseline fluorescence level of each cell, *F*_0_(*t*), was estimated as the 20th percentile of the intensity distribution of *F*(*t*) in a temporal interval of 10 s centered in *t* and activity traces *F*(*t*) were then baselined to obtain Δ*F*/*F*_0_ = [*F*(*t*) - *F*_0_(*t*)]/*F*_0_(*t*) signals (*29*). Δ*F*/*F*_0_ signals were used for all analyses described hereon and will be referred to as “traces”. The peak SNR of Δ*F*/*F*_0_ traces was computed as *max*(Δ*F*/*F*_0_)/*noise*, where *max()* is the maximum value and *noise* was defined as the standard deviation (SD) of the distribution of trace values below the 25th percentile of the distribution of the entire trace (*29*). Pearson’s correlation between traces was computed with the Python function *numpy.corrcoef*. To assess real distances of cells from the center of the FOV or intercellular distances between pairs of cells, we first determined the coordinates in pixels of the centroids of the bounding boxes identified by CITE-ON and built a binary image in which the intensity value of pixels corresponding to box centroids was set to 1 for each FOV. Next, we applied to binary images the appropriate warping transformation (*21*) that was previously obtained with grid calibration measurements with acquisition parameters corresponding to the ones used for in vivo recordings (see paragraph “Calibration grid” above and table S3) using a custom script written in MATLAB (R2024a). Warped centroid images were used to extract the coordinates of cell location within the FOV in microns using the pixel size value reported in table S3. The same warping transformation was applied to average projection images of representative FOVs displayed in the figures.

#### HD tuning analysis

Analysis of HD tuning was carried out using custom code written in Python 3.7. The instantaneous value of the kinematic variable “allocentric HD” was defined as described above. The allocentric HD was calculated for each frame of the behavioral camera and then averaged across four consecutive frames in order to obtain the allocentric HD value to be associated with each frame of the MINI2P. HD tuning curves of recorded cells were obtained from Δ*F*/*F*_0_ traces and behavioral data by: (i) binning the round angle into 20 equal angular bins (bin width 18°); (ii) binning the time points *t* (MINI2P frames) based on the corresponding HD value belonging to a certain angular bin; (iii) computing the mean Δ*F*/*F*_0_ value of frames associated to each angular bin. Resulting HD tuning curves were circularly smoothed using a Gaussian filter (σ = 2). To determine significant HD tuning, we used two different metrics applied to circular tuning curves, the Rayleigh vector length (*46*) and the modulation depth (*30*), defined by Eqs. (1) and (2), respectively.

Rayleigh vector length=(πn sin(πn))·∑j=1nfφje−iφj∑j=1nfφj (1)

where *n* is the number of angular bins (*n* = 20, see above), φ*_j_* and $f_{\varphi_{j}}$ are the *j*-th direction in radians (central value of the *j*-th angular bin) and the Δ*F*/*F_j_* value for the *j*-th angular bin in the tuning curve, respectively, with *j* = 1, .., *n*.

$$\text{Modulation depth}=\frac{\Delta F/F_{\max}-\Delta F/F_{\min}}{\Delta F/F_{\text{mean}}}$$ (2)

where Δ*F*/*F_max_*, Δ*F*/*F_min_* and Δ*F*/*F_mean_* are maximal, minimal, and mean Δ*F*/*F*_0_ values of the tuning curve, respectively. For each metric and each cell, we used a circular permutation non parametric significance testing: the entire calcium trace was shifted 1000 times by a random time interval (minimum shift, 20 s; maximum shift, duration of recording - 20 s) compared to the behavior (time trace of HD values) to obtain shuffled fluorescence traces. Then, HD tuning curves and metric values were computed for all shuffled traces as described before. A cell was considered significantly tuned for HD based on Rayleigh length or modulation depth criterion if the metric value obtained for the real tuning curve was higher than the 95th percentile of the null distribution of metric values computed from circularly permutated traces. This procedure preserved the temporal structure of cell calcium activity traces, while disrupting the association between neuronal calcium dynamics and animal behavior. We evaluated the tuning strength of HD cells by computing the tuning effect size, defined as the difference between the metric value of the cell tuning curve and the 95th percentile of the null distribution divided by the SD of the null distribution. To determine the directionality of tuning, we fitted a von Mises mixture model to tuning curves of HD cells, as done in (*47*), using the Python method *scipy.stats.vonmises.pdf*. The probability density function of a von Mises distribution centered at mean angle μ with concentration k is reported in Eq. (3),

$$f_{\mathrm{vm}}\left(x|\mu ,k\right)=\frac{e^{k\text{cos}\left(x-\mu \right)}}{2\pi I_{0}\left(k\right)}$$ (3)

where *I*_0_(k) is the modified Bessel function of the first kind of order 0. To generalize in case of multimodal directional tuning, we used a weighted sum of multiple von Mises distributions as follows

$$f_{\mathrm{vmm}}\left(x|\mu ,k,\pi \right)=\sum_{j=1}^{M}\pi_{j}f_{\mathrm{vm}}\left(x|\mu_{j},k_{j}\right)$$ (4)

where π*_j_* is the weight of the *j*-th model in the mixture model of order *M*, with *M* number of von Mises distributions in the mixture model and j = 1, .., *M*. The preferred HD direction was defined as the mean angle μ for a single von Mises distribution or the parameter μ associated with the highest peak in case of multiple distributions. The tuning width was estimated as the FWHM of the fitted tuning curve. The spatial spread of a neuronal population within the FOV was defined as the median distance of a cell from its first neighbor. Since numerosity of HD cells and of all cells were different, we performed a random subsampling of all cells to match the numerosity of the HD cells before computing the median for each FOV.

### Histology

Mice were deeply anesthetized with ketamine-xylazine and transcardially perfused with PBS and 4% paraformaldehyde (PFA). Brains were harvested, fixed overnight at 4°C in 4% PFA, and thereafter kept in sucrose (30% v/v in PBS) for sectioning of coronal slices (thickness, 50 μm) at the microtome. Brain sections were either processed immediately or kept in anti-freezing medium at −20°C for later use. Slices selected for histological analyses (AP direction range, −1.4 mm/−1.9 mm from bregma to include the microendoscope implantation site) were washed with PBS and incubated for 30 min with Hoechst 33342 (dilution, 1:300 in PBS) at room temperature to stain cell nuclei. Samples were then mounted on glass slides using 1,4diazabicyclo- (2,2,2) octane (DABCO)-based antifade mounting medium and finally coverslipped. Dual-channel fluorescence images (blue emission for nuclear staining and green emission for endogenous jGCaPM7f signal) were acquired with an inverted fluorescence microscope (Axio Observer, ZEISS, Oberkochen, Germany) equipped with a 10x air objective. Sections from all animals were inspected to verify correct microendoscope implantation over the VPM.

### Statistics

Statistical analyses were carried out using the Python library *scipy.stats* (https://docs.scipy.org/doc/scipy/reference/stats.html). Sample numerosity and number of experimental replicates were stated in each figure caption. Data were presented as mean values ± standard error of the mean (SEM) or ± SD, as specified in figure captions. Data distributions were shown with violin or scatter plots, where the median was indicated by a horizontal line and a numerical value in the graph. In violin plots, the minimum and maximum values of the distribution were also indicated with horizontal lines. Normality of data distributions was assessed with Shapiro-Wilk normality test. *P* values ≥ 0.05 were considered not significant, n.s., and we used the following symbols to indicate statistical significance in figures: *P value < 0.05; **P value < 0.01; ***P value < 0.001. To test the statistical difference between normally distributed dependent samples, we used the paired *t* test. To test the statistical difference between non-normally distributed samples, we used Mann-Whitney U or Wilcoxon test for independent or dependent samples, respectively. Linear fitting was performed using *scipy.stats.linregress*. To test the statistical difference of slopes from zero, we pulled the data from all subjects and performed permutation tests using *scipy.stats.permutation_test* with null distributions built by computing the linear regression of multiple permutations of data (number of resamples, 10^4^ or 10^5^ for *n* < 500 or *n* ≥ 500, respectively, permutation type: ‘pairings’).

## Supplementary Materials

### The PDF file includes:

Figs. S1 to S8 Tables S1 to S4 Legends for movies S1 and S2 Legends for data S1 to S12 References
