# Supplementary material for: Extended field-of-view two-photon imaging in the deep brain of freely behaving mice using lightweight microfabricated corrective optics
Source: Sci Adv. 2026 Oct 16;12(42):eaei5334. doi: 10.1126/sciadv.aei5334 (PMC13674513; doi:10.1126/sciadv.aei5334)
Supplement: Supplementary file 1 [file sciadv.aei5334_sm.pdf]

Science Advances
AAAS

Supplementary Materials for

Extended field-of-view two-photon imaging in the deep brain of freely behaving mice using lightweight microfabricated corrective optics

Chiara Nardin *et al.*

Corresponding author: Tommaso Fellin, tommaso.fellin@iit.it

Sci. Adv. **12**, eaei5334 (2026)
DOI: 10.1126/sciadv.aei5334

The PDF file includes:

Figs. S1 to S8
Tables S1 to S4
Legends for movies S1 and S2
Legends for data S1 to S12
References

Other Supplementary Material for this manuscript includes the following:

Movies S1 and S2
Data S1 to S12

Fig. S1.

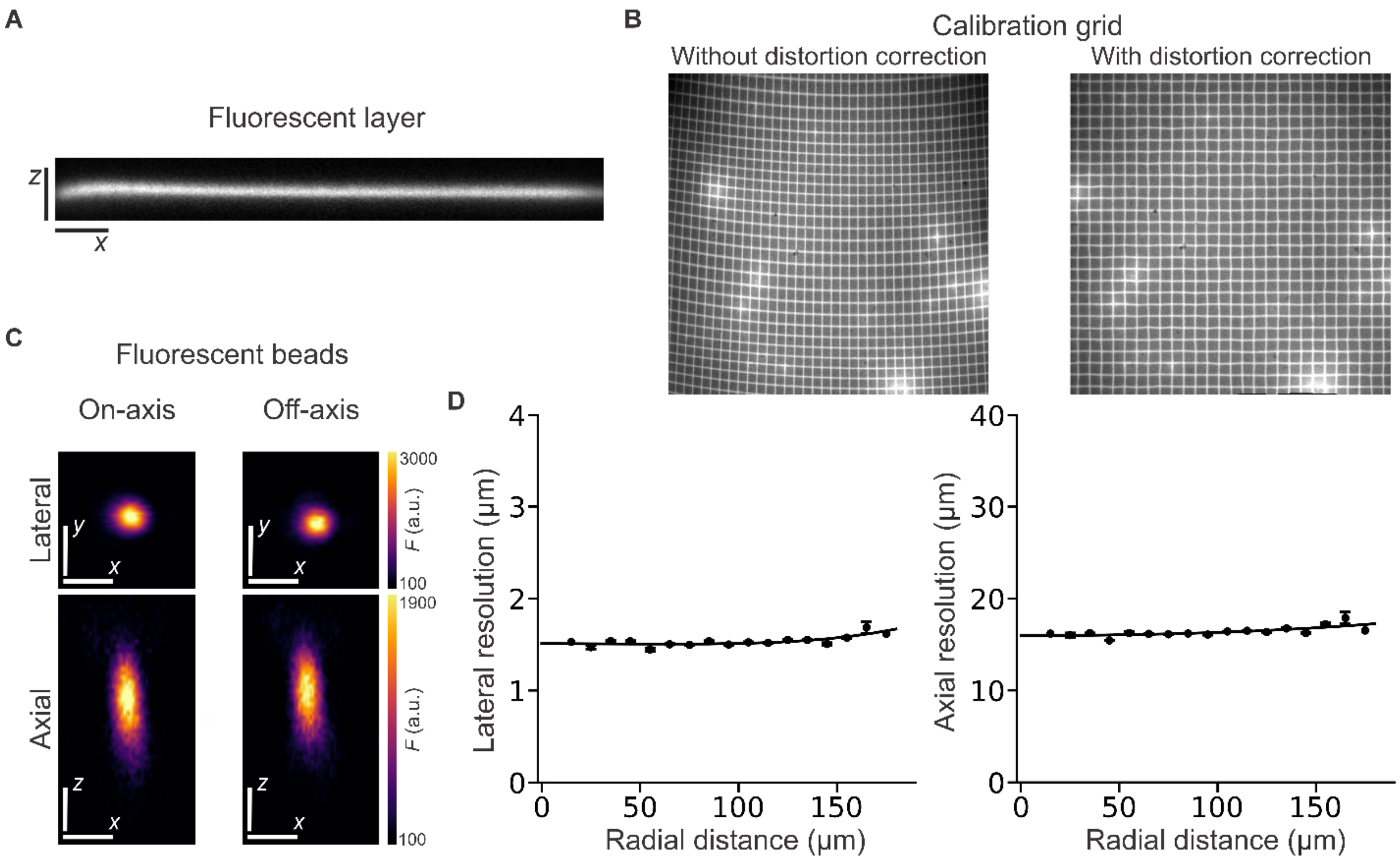

Optical characterization of MINI2P without GRIN microendoscope. (**A**) x (horizontal), z (vertical) intensity projection of a z-stack acquired through a homogeneous subresolved fluorescent layer. The MINI2P was coupled to a 3x air micro-objective (see Materials and Methods). Scale bars: 50 pixels. (**B**) Maximum intensity projection of a z-stack acquired through a fluorescent grid (spacing, 10 µm) before (left) and after (right) applying the algorithm for correction of FOV distortion (*21*). (**C**) Representative x,y (Lateral, top) and x,z (Axial, bottom) average intensity (*F*) projections of z-stacks acquired through a subresolved fluorescent bead. Left, On-axis, radial distance d from the optical axis < 20 µm. Right, Off-axis, radial distance d > 100 µm. Scale bars: x and y, 3 µm; z, 10 µm. (**D**) Lateral (left) and axial (right) resolution as a function of the radial distance (n = 92 beads). Solid lines are quartic functions fitting experimental data (see Table S1).

Fig. S2.

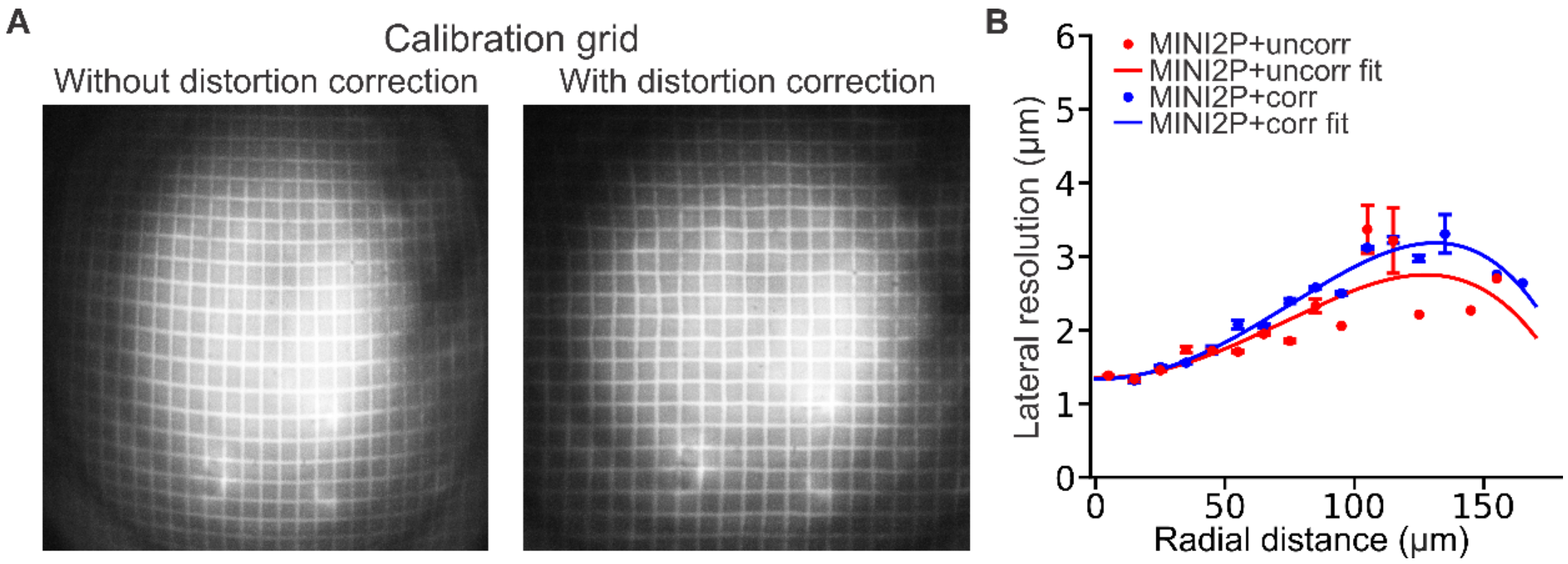

Optical characterization of MINI2P coupled to 4.1 mm-long GRIN microendoscopes. (**A**) Maximum intensity projection of a z-stack acquired through a fluorescent grid (spacing, 10 μm). Images are taken using the MINI2P coupled with a corrected microendoscope of 4.1 mm length and used to correct FOV distortion. An image of the grid is shown before (left) and after (right) the application of the algorithm for correction of FOV distortion (*21*). (**B**) Lateral resolution as a function of the radial distance for MINI2P coupled to uncorrected (red, MINI2P + uncorr) or corrected probes (blue, MINI2P + corr). Data are mean values ± s.e.m. from $n = 3$ experiments. Solid lines are quartic functions fitted to data (see Table S1).

Fig. S3.

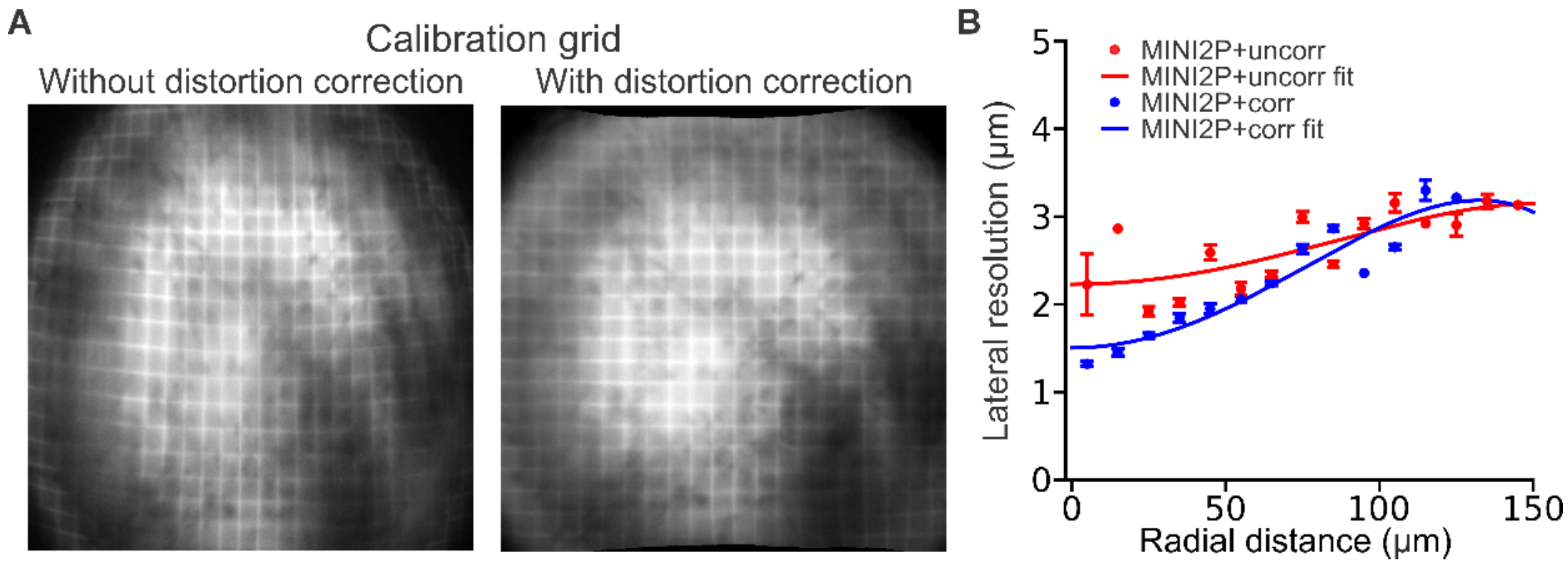

Optical characterization of MINI2P coupled to 6.4 mm-long GRIN microendoscopes. (**A**) Maximum intensity projection of a z-stack acquired through a fluorescent grid (spacing, 10 µm). Images are taken using the MINI2P coupled with a corrected microendoscope of 6.4 mm length and used to correct FOV distortion. An image of the grid is shown before (left) and after (right) the application of the algorithm for correction of FOV distortion (*21*). (**B**) Lateral resolution as a function of the radial distance for MINI2P coupled to uncorrected (red, MINI2P + uncorr) or corrected probes (blue, MINI2P + corr). Data are mean values ± s.e.m. from n = 3 experiments. Solid lines are quartic functions fitted to data (see Table S1).

Fig. S4.

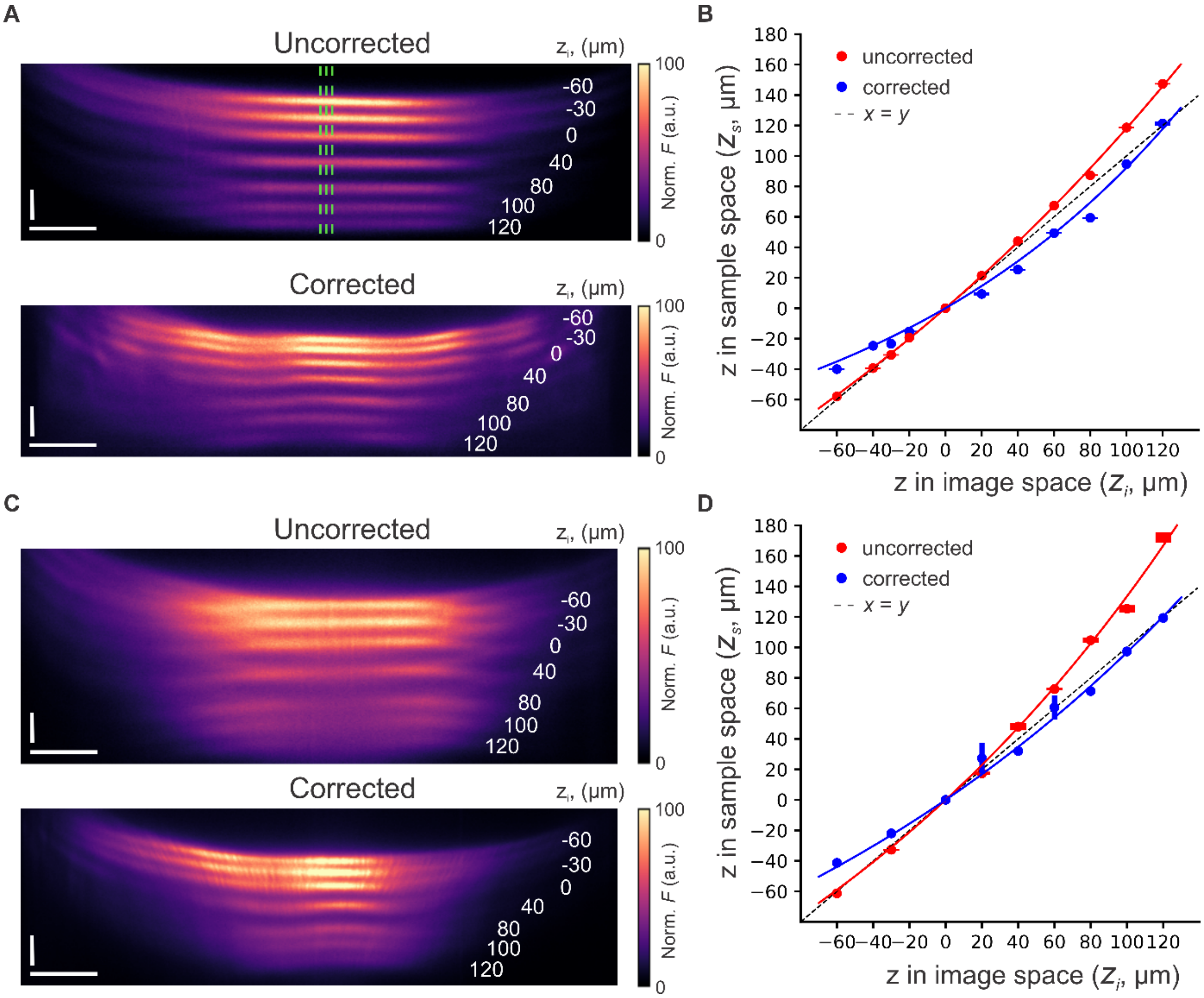

Volumetric imaging using the MINI2P coupled with uncorrected and corrected microendoscopes. (**A**) Normalized x,z average intensity projections of z-stacks acquired through a homogeneous subresolved fluorescent layer using the MINI2P coupled with a 4.1 mm-long uncorrected (top) or corrected (bottom) microendoscope. Each stack is acquired at different axial positions in the image space (numbers on the right side of the image, z_i, see the inset in Fig. 1C for definition). Stacks acquired at different z_i positions are shown overlaid. $z_i = 0$ corresponds to the z-axis position of the focal plane in the image space at which the FOV of the corrected microendoscope was maximally enlarged (nominal focal plane). Scale bars: 50 µm. (**B**) z-axis position of the focal plane in the sample space (z_s, see the inset in Fig. 1C for definition) for MINI2P coupled with a 4.1 mm-long uncorrected (red) or corrected (blue) microendoscope as a function of z_i for the data shown in (A). $z_s = 0$ corresponds to the z-axis position of the fluorescent layer imaged at $z_i = 0$. Data are mean values ± s.e.m. of local maxima of the fluorescence intensity profiles obtained from the overlay images shown in (A) along three vertical segments located near the center of the FOV (green dashed lines in A, top image). The solid curves are exponential functions $f(x) = a(e^{bx}-1)$ fitted to experimental data. The black dashed line represents the diagonal. (**C, D**) Same as (A, B) for the MINI2P coupled with the 6.4 mm-long microendoscope.

Fig. S5.

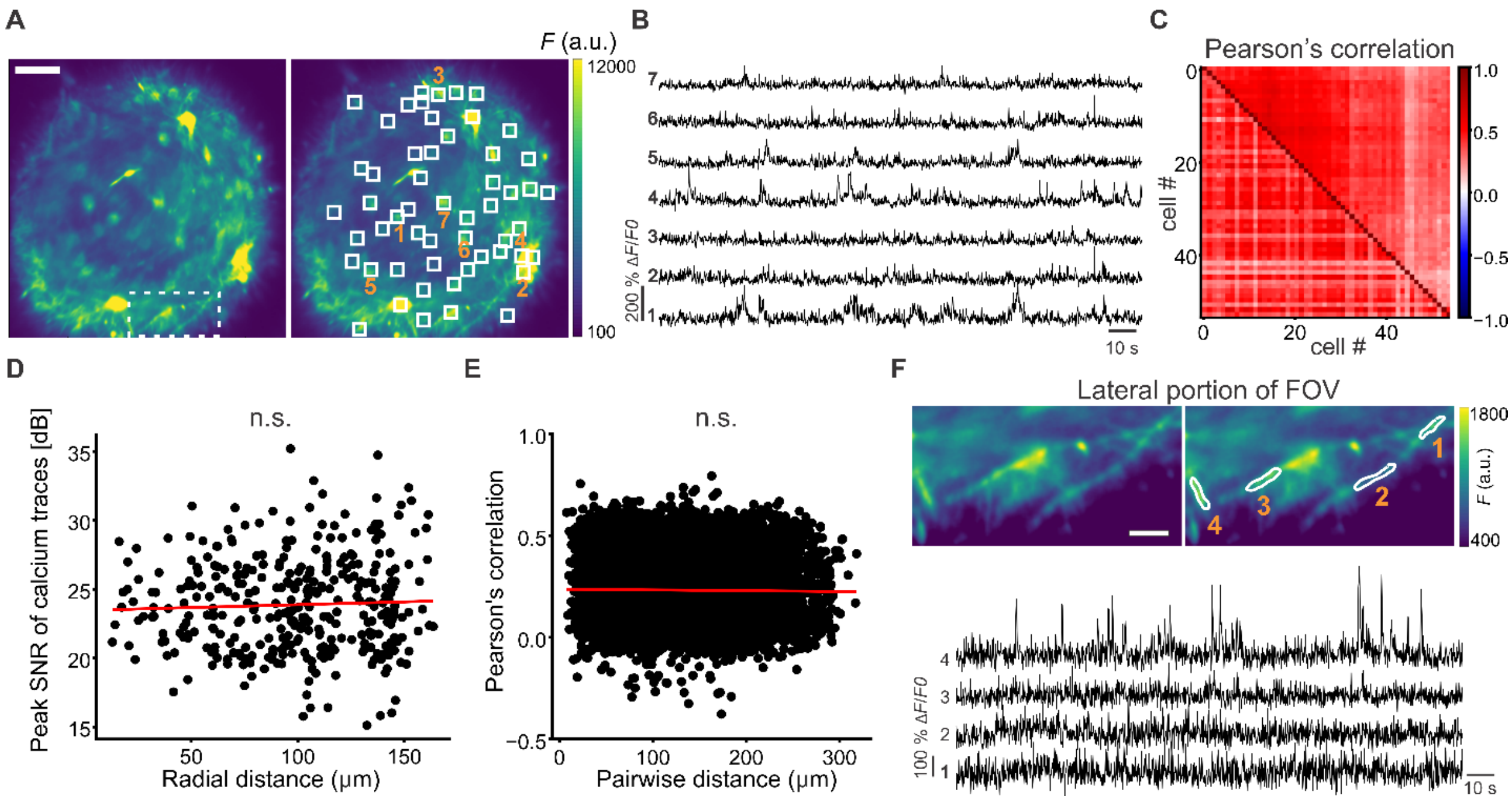

Extended FOV imaging of thalamic activity during open field navigation: raw data. (**A**) Left: representative FOV. Scale bar: 50 μm. Right: detected cell identities are surrounded by white rectangles (n = 54 cells). Displayed images are averages across all frames of the t-series from the same experiment shown in Fig. 4D but on non-denoised data. (**B**) Fluorescence traces for cellular identities marked with ordinal numbers in (A). (**C**) Lower-left triangle: Pearson's correlation matrix for detected cells in (A). Upper-right triangle: corresponding hierarchical clustering. (**D**) Peak SNR of fluorescence traces over radial distance (n = 353 cells from m = 8 FOVs). Red line: linear regression. Slope being different from 0 was tested with permutation test; n.s., not significant, P value = 0.39. (**E**) Pearson's correlation between pairs of neurons as a function of intercellular distance (n = 8581 pairs from m = 8 FOVs). Red line: linear regression. Slope being different from 0 was tested with a permutation test; P value = 0.19. (**F**) Top left: zoom-in view of the portion of the FOV indicated in (A) with the white dashed rectangle. Scale bar: 20 μm. Top right: white solid contours indicate segmented portions of neuronal processes. Bottom: calcium signals extracted from segmented neuronal processes. Displayed images are averages across all frames of the t-series from the same experiment shown in Fig. 4I but on non-denoised data.

Fig. S6.

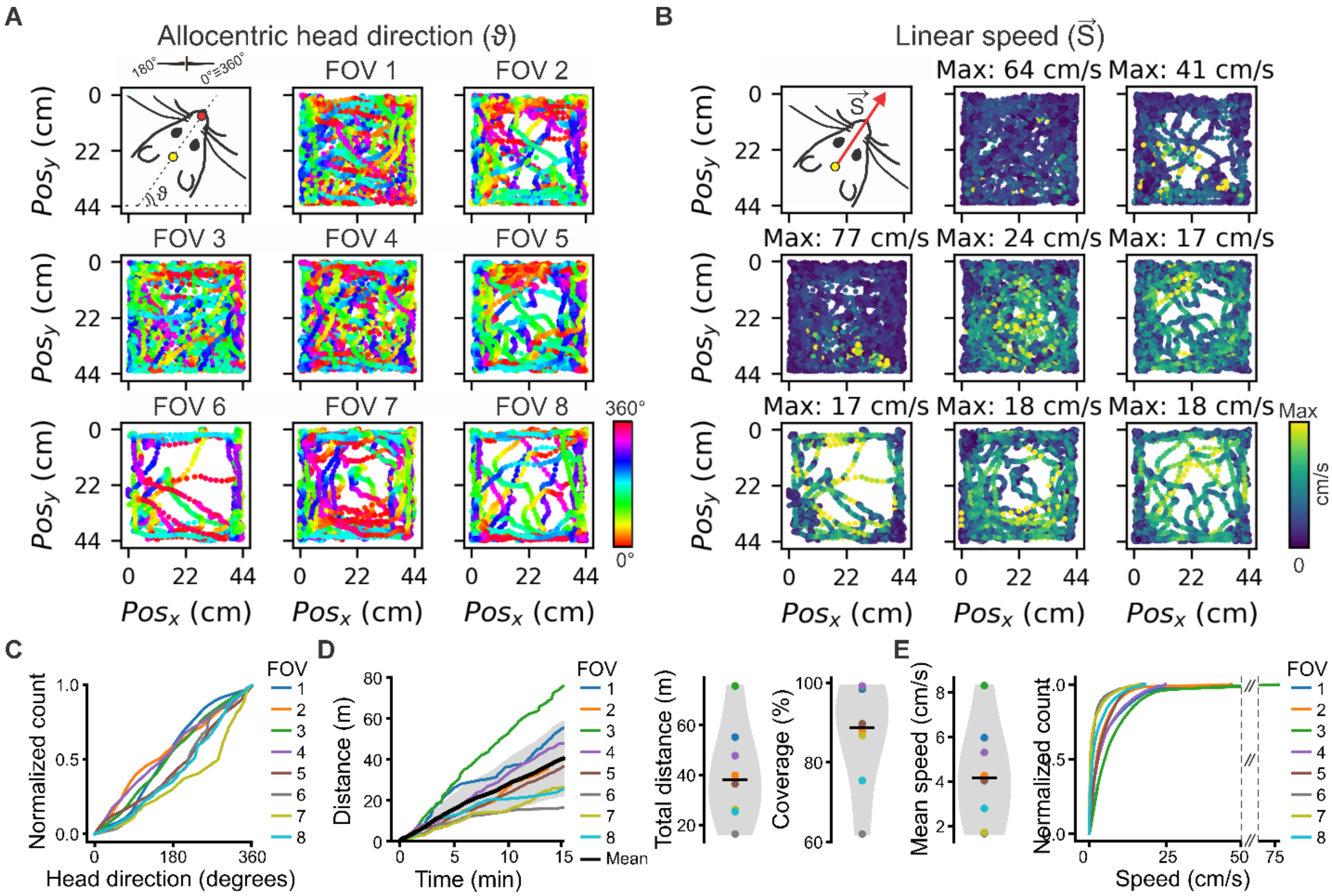

Behavioral parameters during open field navigation and MINI2P deep brain imaging. (**A**) Frame-by-frame trajectories followed by mice during open field spatial navigation in the square arena are shown color-coded based on instantaneous allocentric HD (graphical definition in top left square, see also Materials and Methods) for n = 8 FOVs from m = 6 mice. (**B**) Same as (A) with color-code based on mouse linear speed. The maximum speed reached during the session is indicated above each plot. (**C**) Cumulative distribution of HD. The color-code indicates individual FOVs. (**D**) Left: cumulative distance covered across each recording session (15 min). The color-code indicates individual FOVs. The black line and the gray shaded areas represent the mean and s.d. across FOVs. Center: total traveled distance within a recording session for each FOV. The black horizontal line marks the median across FOVs. Right: behavioral coverage of the area of the arena during open field navigation. The black horizontal line marks the median across FOVs. (**E**) Left: mean mouse speed across FOVs. The color-code indicates individual FOVs. The black horizontal line marks the median across FOVs. Right: Cumulative distribution of running speed for each FOV.

Fig. S7.

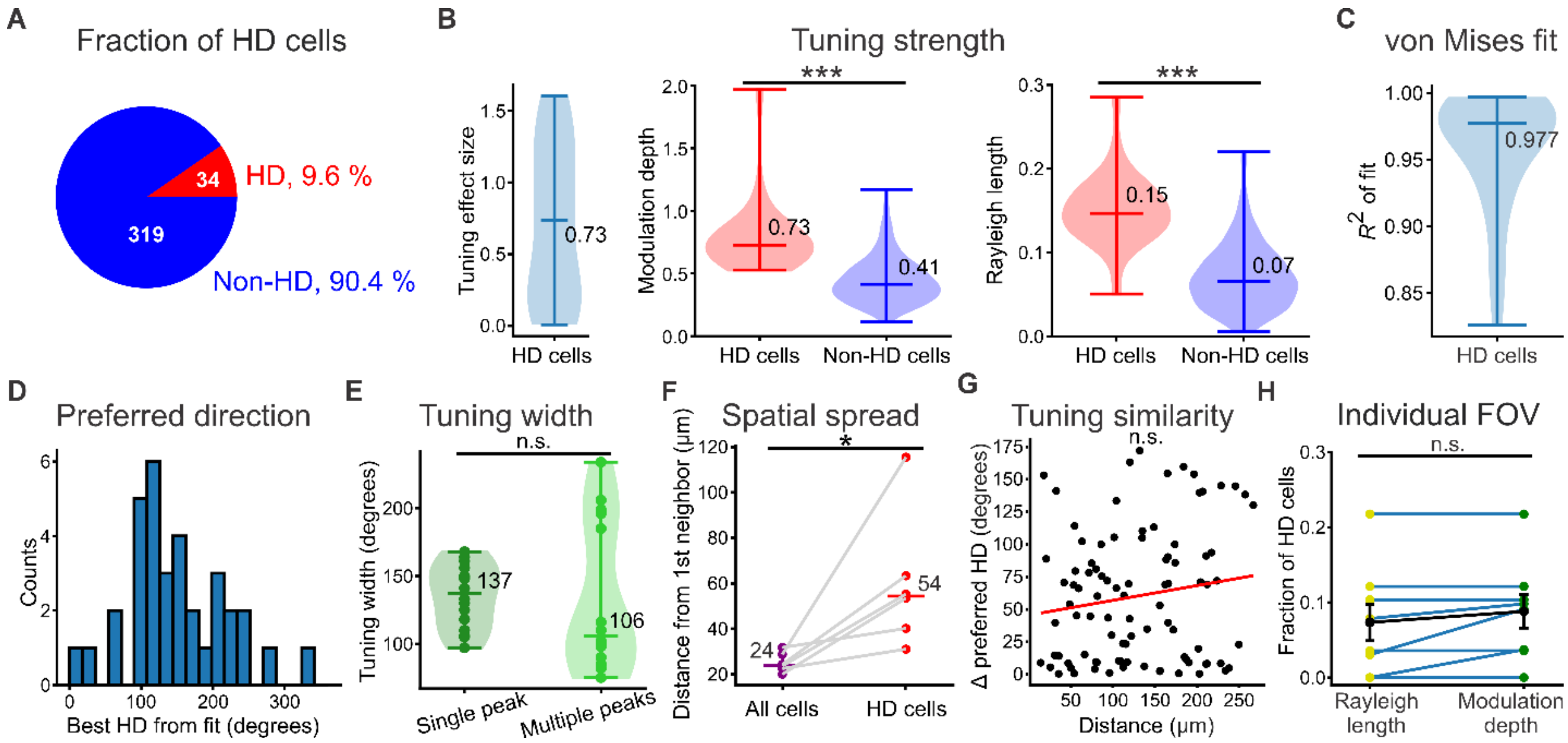

HD tuning in the VPM during open field navigation: results based on quantification of modulation depth. (**A**) Fraction of HD cells over all imaged VPM neurons (n = 353 cells, m = 8 FOVs, same data presented in Fig. 5) based on the modulation depth criterion. (**B**) Left: distribution of effect size of tuning strength for HD cells identified with the modulation depth criterion. Horizontal lines mark max/min values and median across HD cells. Center: distribution of modulation depth values for HD (red) and non HD cells (blue). $P < 10^{-14}$, Mann-Whitney U test. Right: distribution of Rayleigh length values for HD (red) and non HD cells (blue). $P < 10^{-16}$, Mann-Whitney U test. (**C**) Distribution of R^2 values of von Mises models fitted to the tuning curves of HD cells. (**D**) Distribution of preferred HD for HD cells. Median: 134°. (**E**) Tuning width for HD cells with a single (n = 16) or multiple peaks (n = 18) identified by the von Mises model. P = 0.27, Mann-Whitney U test. (**F**) Median distance from first neighbor across cells and HD cells for each FOV (n = 6 FOVs with at least 2 HD cells per FOV). P = 0.040, paired t-test. (**G**) Angular difference between preferred HD of HD tuned cell pairs as a function of intercellular distance (n = 92 pairs from m = 8 FOVs). Red line: linear regression. Slope being different from 0 was tested with permutation test; P = 0.15. (**H**) Fraction of cells significantly tuned for HD in n = 8 individual FOVs, identified using either the Rayleigh length (yellow) or the modulation depth (green) criterion. Black dots and lines mark the mean values ± s.e.m., P value = 0.11, paired t-test.

Fig. S8.

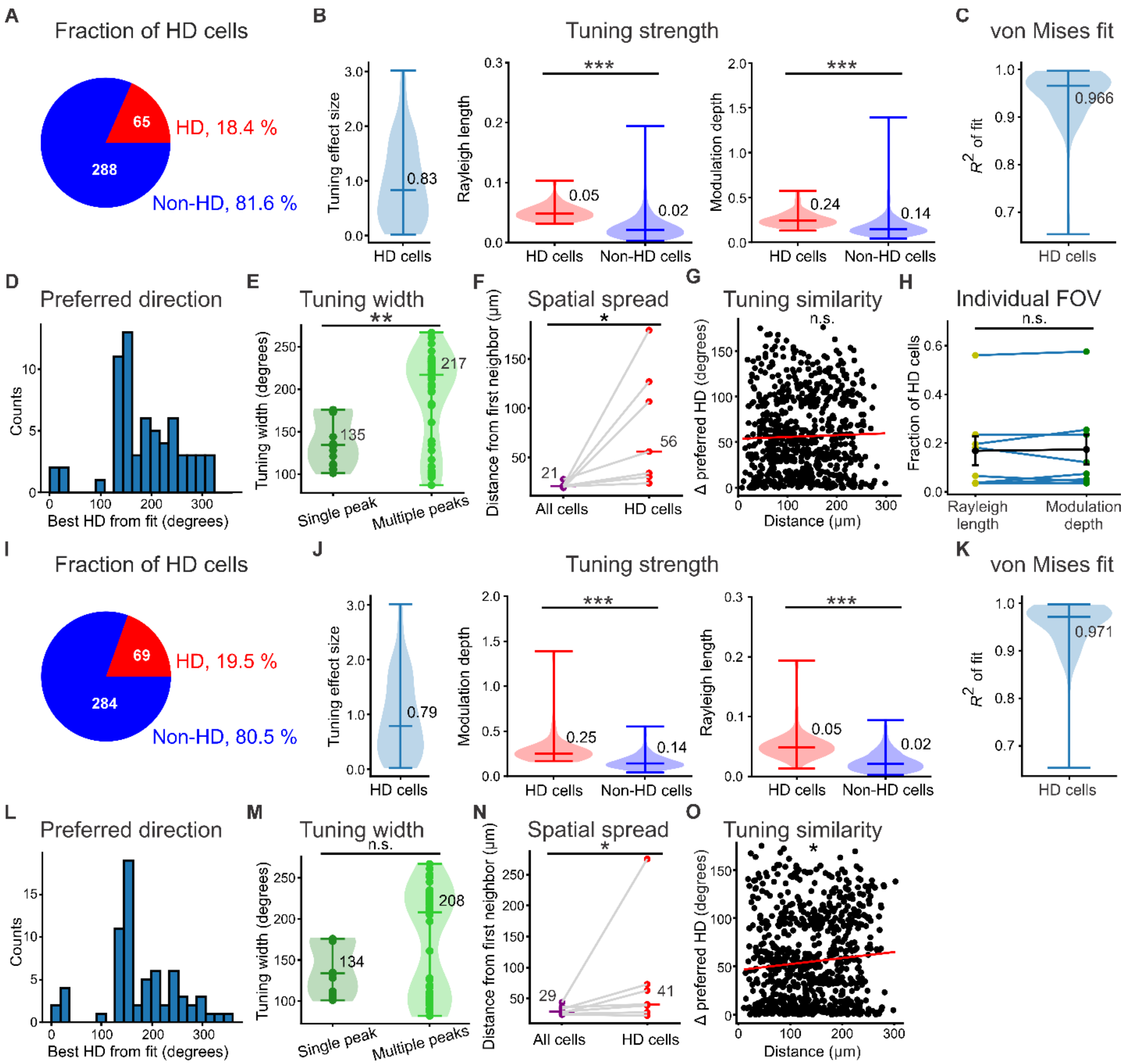

HD tuning analysis performed on raw data. (**A**) Fraction of HD cells over all imaged VPM neurons (n = 353 cells, m = 8 FOVs) based on the Rayleigh length criterion. (**B**) Left: distribution of effect size of tuning strength for HD cells identified with the Rayleigh length criterion. Horizontal lines mark max/min values and median across HD cells. Center: distribution of Rayleigh length values for HD (red) and non HD cells (blue). $P < 10^{-24}$, Mann-Whitney U test. Right: distribution of modulation depth values for HD (red) and non HD cells (blue). $P < 10^{-16}$, Mann-Whitney U test. (**C**) Distribution of R^2 values of von Mises models fitted to the tuning curves of HD cells. (**D**) Distribution of preferred HD for HD cells. Median: 180°. (**E**) Tuning width for HD cells with a single (n = 18) or multiple peaks (n = 47) identified by the von Mises model. P = 0.001, Mann-Whitney U test. (**F**) Median distance from first neighbor across all cells and across HD cells for each FOV (n = 7 FOVs with at least 2 HD cells per FOV). P = 0.016, Wilcoxon test. (**G**) Angular difference between preferred HD of HD tuned cell pairs as a function of intercellular

distance (*n* = 737 pairs from *m* = 8 FOVs). The red line represents linear regression. Slope being different from 0 was tested with permutation test; $P = 0.43$. (**H**) Fraction of cells significantly tuned for HD in *n* = 8 individual FOVs, identified using either the Rayleigh length (yellow) or the modulation depth (green) criterium. Black dots and lines mark the mean values ± s.e.m. $P = 0.75$, Wilcoxon test. (**I** to **O**) Same as (**A** to **G**) for HD tuning analysis based on the modulation depth criterion. (**J**) Center: $P < 10^{-22}$, Mann-Whitney U test. Right: $P < 10^{-23}$, Mann-Whitney U test. (**L**) Median: 152°. (**M**) HD cells with a single peak, *n* = 15, or multiple peaks, *n* = 54. $P = 0.26$, Mann-Whitney U test. (**N**) $P = 0.031$, Wilcoxon test. (**O**) *n* = 803 pairs from *m* = 8 FOVs; $P = 0.011$, permutation test.

Table S1.

<table>
<tr><td colspan="6">MINI2P</td></tr>
<tr><td colspan="3">Lateral resolution (μm)
$f(x) = ax^4 + bx^2 + c$</td><td colspan="3">Axial resolution (μm)
$f'(x) = a'x^4 + b'x^2 + c'$</td></tr>
<tr><td>a</td><td colspan="2">$(2.1 \pm 1.1)\cdot 10^{-10}$</td><td>$a'$</td><td colspan="2">$(0.23 \pm 1.2)\cdot 10^{-9}$</td></tr>
<tr><td>b</td><td colspan="2">$(-1.9 \pm 3.3)\cdot 10^{-6}$</td><td>$b'$</td><td colspan="2">$(3.3 \pm 3.6)\cdot 10^{-5}$</td></tr>
<tr><td>c</td><td colspan="2">1.509 ± 0.018</td><td>c'</td><td colspan="2">16.00 ± 0.19</td></tr>
<tr><td>χ^2</td><td colspan="2">1.00</td><td>χ^2</td><td colspan="2">1.00</td></tr>
<tr><td colspan="6">MINI2P coupled to 4.1 mm-long GRIN microendoscopes</td></tr>
<tr><td colspan="3">Lateral resolution (μm)
$f(x) = ax^4 + bx^2 + c$</td><td colspan="3">Axial resolution (μm)
$f'(x) = a'x^4 + b'x^2 + c'$</td></tr>
<tr><td></td><td>Uncorrected</td><td>Corrected</td><td></td><td>Uncorrected</td><td>Corrected</td></tr>
<tr><td>a</td><td>$(-5.3 \pm 1.9)\cdot 10^{-9}$</td><td>$(-62.5 \pm 5.7)\cdot 10^{-10}$</td><td>$a'$</td><td>$(73.0 \pm 1.9)\cdot 10^{-8}$</td><td>$(-46.3 \pm 8.9)\cdot 10^{-9}$</td></tr>
<tr><td>b</td><td>$(17.3 \pm 4.3)\cdot 10^{-5}$</td><td>$(21.5 \pm 1.5)\cdot 10^{-5}$</td><td>$b'$</td><td>$(14.1 \pm 4.3)\cdot 10^{-4}$</td><td>$(16.8 \pm 2.4)\cdot 10^{-4}$</td></tr>
<tr><td>c</td><td>1.35 ± 0.18</td><td>1.332 ± 0.072</td><td>c'</td><td>17.7 ± 1.7</td><td>12.1 ± 1.1</td></tr>
<tr><td>χ^2</td><td>1.00</td><td>1.00</td><td>χ^2</td><td>0.989</td><td>0.999</td></tr>
<tr><td colspan="6">MINI2P coupled to 6.4 mm-long GRIN microendoscopes</td></tr>
<tr><td colspan="3">Lateral resolution (μm)
$f(x) = ax^4 + bx^2 + c$</td><td colspan="3">Axial resolution (μm)
$f'(x) = a'x^4 + b'x^2 + c'$</td></tr>
<tr><td></td><td>Uncorrected</td><td>Corrected</td><td></td><td>Uncorrected</td><td>Corrected</td></tr>
<tr><td>a</td><td>$(-1.8 \pm 2.0)\cdot 10^{-9}$</td><td>$(-5.5 \pm 2.9)\cdot 10^{-9}$</td><td>$a'$</td><td>$(16.0 \pm 4.0)\cdot 10^{-8}$</td><td>$(-6.2 \pm 6.2)\cdot 10^{-8}$</td></tr>
<tr><td>b</td><td>$(8.0 \pm 4.1)\cdot 10^{-5}$</td><td>$(19.2 \pm 4.4)\cdot 10^{-5}$</td><td>$b'$</td><td>$(-13.3 \pm 7.9)\cdot 10^{-4}$</td><td>$(19.1 \pm 9.2)\cdot 10^{-4}$</td></tr>
<tr><td>c</td><td>2.23 ± 0.14</td><td>1.50 ± 0.12</td><td>c'</td><td>33.9 ± 2.8</td><td>17.0 ± 2.5</td></tr>
<tr><td>χ^2</td><td>1.00</td><td>1.00</td><td>χ^2</td><td>0.742</td><td>0.725</td></tr>
</table>

Numerical coefficients of quartic functions fitting lateral and axial resolution data for MINI2P coupled to GRIN microendoscopes. Average ± s.d. of the parameters of quartic function fitting experimental measurements of the lateral (left) and axial (right) resolution using MINI2P in the following conditions: i) not coupled to a GRIN microendoscope (top, see Fig. S1D); coupled to uncorrected and corrected microendoscopes based on the 4.1 mm-long GRIN lens (middle, see Fig. S2B and Fig. 1F); coupled to uncorrected and corrected microendoscopes based on the 6.4 mm-long GRIN rod (bottom, see Fig. S3B and Fig. 2B). Note that coefficients c and c' correspond to the lateral and axial resolution of the optical system on-axis. χ^2 values for the goodness of fit are indicated for each curve.

Table S2.

MINI2P + 4.1 mm-long microendoscope		MINI2P + 6.4 mm-long microendoscope	
**Effective FOV radius** (μm) (threshold = 20 μm)	**Uncorrected:** 40 **Corrected:** 74	**Effective FOV radius** (μm) (threshold = 20 μm)	**Uncorrected:** n.a. **Corrected:** 41
**Effective FOV radius** (μm) (threshold = 19.5 μm)	**Uncorrected:** 35 **Corrected:** 71	**Effective FOV radius** (μm) (threshold = 37.3 μm)	**Uncorrected:** 102 **Corrected:** 128
**Fold increase in radius**	2.02	**Fold increase in radius**	1.26
**Fold increase in area**	4.08	**Fold increase in area**	1.58

FOV enlargement when MINI2P is coupled to corrected microendoscopes. Effective FOV radii (computed with two approaches, see Materials and Methods) and FOV enlargement (see Materials and Methods) of corrected systems compared to uncorrected systems for the 4.1 mm-long (left) and 6.4 mm-long GRIN microendoscope (right). n.a., not applicable.

Table S3.

Acquisition parameters	Sample	MINI2P	MINI2P + 4.1 mm-long microendoscope		MINI2P + 6.4 mm-long microendoscope	
**3x air D0254, zoom = 1, 2048 x 2048 pixels**	Fluorescent beads	-	**Uncorrected**	0.17 µm/pixel	**Uncorrected**	0.31 µm/pixel
			**Corrected**	0.13 µm/pixel	**Corrected**	0.13 µm/pixel
**3x air D0254, zoom = 2, 2048 x 2048 pixels**	Fluorescent beads	0.13 µm/pixel	**Uncorrected**	0.10 µm/pixel	**Uncorrected**	0.19 µm/pixel
			**Corrected**	0.10 µm/pixel	**Corrected**	0.10 µm/pixel
**3x air D0254, zoom = 1, 1024 x 1024 pixels**	Brain slices	-	**Uncorrected**	0.44 µm/pixel	**Uncorrected**	0.44 µm/pixel
			**Corrected**	0.44 µm/pixel	**Corrected**	0.44 µm/pixel
**3x air/glass D0309, zoom = 1, 512 x 512 pixels**	In vivo VPM imaging	-	**Corrected**	0.72 µm/pixel	-	-

Pixel sizes of MINI2P data under the different experimental conditions. Data refer to real pixel sizes after calibration of FOV distortion (*21*).

Table S4.

Reference	Miniaturized multiphoton microscope type and λ (nm)	Deep brain region	Implanted optical element	Size of implanted optical element (mm)	PSF size on-axis (µm)	FOV area (µm^2)	Experimental paradigm
(*21*)	MINI2P-L<br>2P λ: 920	Mouse hippocampal CA1	GRIN lens	Diameter: 1<br>Length: 4.3	n.a.	420 × 420	Free-foraging open field navigation
(*22*)	FHIRM-TPM 3.0-U<br>2P λ: 920	Mouse dorsal hippocampal CA1	GRIN lens	Diameter: 1<br>Length: 4	Lateral: 1.05 ± 0.01<br>Axial: 11 ± 0.12	148 ×120	Head-fixation
(*48*)	TM-MINI2P<br>2P λ: 920	Mouse prefrontal cortex	GRIN lens	Diameter: 1<br>Length: 4.38	n.a.	500 × 500	Open field navigation with auditory stimulation
(*49*)	millimeter FOV m2PM<br>2P λ: 920	Mouse dorsal hippocampal CA1	GRIN lens	Diameter: 1<br>Length: 3.92	n.a.	900 × 788	Open field navigation
This work	MINI2P-L<br>2P λ: 920	Mouse ventropostero-medial thalamic nucleus (VPM)	GRIN lens	Diameter: 0.5<br>Length: 4.1	Lateral: 1.33 ± 0.07<br>Axial: 12.1 ± 1.1	350 × 350	Open field navigation
(*21*)	MINI2P-L<br>2P λ: 920	Mouse medial entorhinal cortex (MEC)	Right angle microprism	Leg lengths:<br>1.3<br>1.3<br>1.6	n.a.	500 × 500	Free-foraging open field navigation

(*50*)	MINI2P<br>2P λ: 920	Mouse dorsal CA1	Glass plug	Diameter: 1.8<br>Length: 1.5	n.a.	350 × 350	Spatial foraging navigation
(*51*)	MINI2P-L<br>2P λ: 1064	Mouse dorsal CA1	Optical window	Cannula diameter: 3<br>Height: 1.6	n.a.	~ 360 × 360	Open field navigation
(*52*)	2P/3P head-mounted multiplane microscope<br>3P λ: 1300	Mouse visual cortex L5/ posterior parietal cortex L5	None	-	3P Lateral: 1.30 ± 0.03 Axial: 14.8 ± 1.1	450 × 600	Open field navigation/gap-crossing task

Applications of miniaturized multiphoton microscopes for high-resolution imaging of deep (> 450 μm) brain regions in freely moving animals. List of published studies and corresponding experimental conditions. n.a. = not available.

Movie S1.

MINI2P imaging in the VPM of freely moving mice. VPM neurons expressing jGCaMP7f (right movie) during open field navigation (left movie) acquired with MINI2P coupled to the 4.1 mm-long corrected GRIN microendoscope: raw motion-corrected data.

Movie S2.

MINI2P imaging in the VPM of freely moving mice and denoising. VPM neurons expressing jGCaMP7f (right movie) during open field navigation (left movie) acquired with MINI2P coupled to the 4.1 mm-long corrected GRIN microendoscope: denoised motion-corrected data.

Data S1.

Source data file for Fig. 1, panel F.

Data S2.

Source data file for Fig. 2, panel B.

Data S3.

Source data file for Fig. 4, panels E to I.

Data S4.

Source data file for Fig. 5, panels B to H, J and K.

Data S5.

Source data file for Fig. S1, panel D.

Data S6.

Source data file for Fig. S2, panel B.

Data S7.

Source data file for Fig. S3, panel B.

Data S8.

Source data file for Fig. S4, panels B and D.

Data S9.

Source data file for Fig. S5, panels B to F.

Data S10.

Source data file for Fig. S6, panels A to E.

Data S11.

Source data file for Fig. S7, panels A to H.

Data S12.

Source data file for Fig. S8, panels A to O.

REFERENCES

1. K. D. Harris, R. Q. Quiroga, J. Freeman, S. L. Smith, Improving data quality in neuronal population recordings. *Nat. Neurosci.* **19**, 1165–1174 (2016).

2. L. Carrillo-Reid, R. Yuste, Playing the piano with the cortex: Role of neuronal ensembles and pattern completion in perception and behavior. *Curr. Opin. Neurobiol.* **64**, 89–95 (2020).

3. R. Yuste, R. Cossart, E. Yaksi, Neuronal ensembles: Building blocks of neural circuits. *Neuron* **112**, 875–892 (2024).

4. W. Denk, J. H. Strickler, W. W. Webb, Two-photon laser scanning fluorescence microscopy. *Science* **248**, 73–76 (1990).

5. F. Helmchen, W. Denk, Deep tissue two-photon microscopy. *Nat. Methods* **2**, 932–940 (2005).

6. T. W. Chen, T. J. Wardill, Y. Sun, S. R. Pulver, S. L. Renninger, A. Baohan, E. R. Schreiter, R. A. Kerr, M. B. Orger, V. Jayaraman, L. L. Looger, K. Svoboda, D. S. Kim, Ultrasensitive fluorescent proteins for imaging neuronal activity. *Nature* **499**, 295–300 (2013).

7. *H. Dana*, Y. Sun, B. Mohar, B. K. Hulse, A. M. Kerlin, J. P. Hasseman, G. Tsegaye, A. Tsang, A. Wong, R. Patel, J. J. Macklin, Y. Chen, A. Konnerth, V. Jayaraman, L. L. Looger, E. R. Schreiter, K. Svoboda, D. S. Kim, High-performance calcium sensors for imaging activity in neuronal populations and microcompartments. *Nat. Methods* **16**, 649–657 (2019).

8. Y. Zhang, M. Rózsa, Y. Liang, D. Bushey, Z. Wei, J. Zheng, D. Reep, G. J. Broussard, A. Tsang, G. Tsegaye, S. Narayan, C. J. Obara, J.-X. Lim, R. Patel, R. Zhang, M. B. Ahrens, G. C. Turner, S. S.-H. Wang, W. L. Korff, E. R. Schreiter, K. Svoboda, J. P. Hasseman, I. Kolb, L. L. Looger, Fast and sensitive GCaMP calcium indicators for imaging neural populations. *Nature* **615**, 884–891 (2023).

9. K. Ohki, S. Chung, Y. H. Ch'ng, P. Kara, R. C. Reid, Functional imaging with cellular resolution reveals precise micro-architecture in visual cortex. *Nature* **433**, 597–603 (2005).

10. K. Ohki, S. Chung, P. Kara, M. Hübener, T. Bonhoeffer, R. C. Reid, Highly ordered arrangement of single neurons in orientation pinwheels. *Nature* **442**, 925–928 (2006).

11. L. Carrillo-Reid, W. Yang, Y. Bando, D. S. Peterka, R. Yuste, Imprinting and recalling cortical ensembles. *Science* **353**, 691–694 (2016).

12. P. Golshani, J. T. Gonçalves, S. Khoshkhoo, R. Mostany, S. Smirnakis, C. Portera-Cailliau, Internally mediated developmental desynchronization of neocortical network activity. *J. Neurosci.* **29**, 10890–10899 (2009).

13. J. E. Miller, I. Ayzenshtat, L. Carrillo-Reid, R. Yuste, Visual stimuli recruit intrinsically generated cortical ensembles. *Proc. Natl. Acad. Sci. U.S.A.* **111**, E4053–E4061 (2014).

14. E. D. Vickers, D. A. McCormick, Pan-cortical 2-photon mesoscopic imaging and neurobehavioral alignment in awake, behaving mice. *eLife* **13**, RP94167 (2024).

15. Y. Masamizu, Y. R. Tanaka, Y. H. Tanaka, R. Hira, F. Ohkubo, K. Kitamura, Y. Isomura, T. Okada, M. Matsuzaki, Two distinct layer-specific dynamics of cortical ensembles during learning of a motor task. *Nat. Neurosci.* **17**, 987–994 (2014).

16. M. Murakami, M. I. Vicente, G. M. Costa, Z. F. Mainen, Neural antecedents of self-initiated actions in secondary motor cortex. *Nat. Neurosci.* **17**, 1574–1582 (2014).

17. W. R. Zipfel, R. M. Williams, W. W. Webb, Nonlinear magic: Multiphoton microscopy in the biosciences. *Nat. Biotechnol.* **21**, 1369–1377 (2003).

18. K. Svoboda, R. Yasuda, Principles of two-photon excitation microscopy and its applications to neuroscience. *Neuron* **50**, 823–839 (2006).

19. W. Zong, R. Wu, M. Li, Y. Hu, Y. Li, J. Li, H. Rong, H. Wu, Y. Xu, Y. Lu, H. Jia, M. Fan, Z. Zhou, Y. Zhang, A. Wang, L. Chen, H. Cheng, Fast high-resolution miniature two-photon microscopy for brain imaging in freely behaving mice. *Nat. Methods* **14**, 713–719 (2017).

20. W. Zong, R. Wu, S. Chen, J. Wu, H. Wang, Z. Zhao, G. Chen, R. Tu, D. Wu, Y. Hu, Y. Xu, Y. Wang, Z. Duan, H. Wu, Y. Zhang, J. Zhang, A. Wang, L. Chen, H. Cheng, Miniature two-photon microscopy for enlarged field-of-view, multi-plane and long-term brain imaging. *Nat. Methods* **18**, 46–49 (2021).

21. W. Zong, H. A. Obenhaus, E. R. Skytøen, H. Eneqvist, N. L. de Jong, R. Vale, M. R. Jorge, M.-B. Moser, E. I. Moser, Large-scale two-photon calcium imaging in freely moving mice. *Cell* **185**, 1240–1256.e30 (2022).

22. R. Wu, C. Zhao, S. Qiu, Y. Zhu, L. Zhang, Q. Fu, Y. Hu, D. Wu, F. Yu, F. Zhou, H. Huang, Y. Zhang, X. Wang, A. Wang, H. Cheng, A versatile miniature two-photon microscope enabling multicolor deep-brain imaging. *Nat. Methods* **22**, 1935–1943 (2025).

23. J. C. Jung, M. J. Schnitzer, Multiphoton endoscopy. *Opt. Lett.* **28**, 902–904 (2003).

24. F. Bortoletto, C. Bonoli, P. Panizzolo, C. D. Ciubotaru, F. Mammano, Multiphoton fluorescence microscopy with GRIN objective aberration correction by low order adaptive optics. *PLOS ONE* **6**, e22321 (2011).

25. C. Wang, N. Ji, Pupil-segmentation-based adaptive optical correction of a high-numerical-aperture gradient refractive index lens for two-photon fluorescence endoscopy. *Opt. Lett.* **37**, 2001–2003 (2012).

26. C. Wang, N. Ji, Characterization and improvement of three-dimensional imaging performance of GRIN-lens-based two-photon fluorescence endomicroscopes with adaptive optics. *Opt. Express* **21**, 27142–27154 (2013).

27. Y. Li, Z. Cheng, C. Wang, J. Lin, H. Jiang, M. Cui, Geometric transformation adaptive optics (GTAO) for volumetric deep brain imaging through gradient-index lenses. *Nat. Commun.* **15**, 1031 (2024).

28. A. Antonini, A. Sattin, M. Moroni, S. Bovetti, C. Moretti, F. Succol, A. Forli, D. Vecchia, V. P. Rajamanickam, A. Bertoncini, S. Panzeri, C. Liberale, T. Fellin, Extended field-of-view ultrathin

microendoscopes for high-resolution two-photon imaging with minimal invasiveness. *eLife* **9**, e58882 (2020).

29. A. Sattin, C. Nardin, S. Daste, M. Moroni, I. Reddy, C. Liberale, S. Panzeri, A. Fleischmann, T. Fellin, Aberration correction in long GRIN lens-based microendoscopes for extended field-of-view two-photon imaging in deep brain regions. *eLife* **13**, RP101420 (2025).

30. T. B. Oram, A. Tenzer, I. Saraf-Sinik, O. Yizhar, E. Ahissar, Co-coding of head and whisker movements by both VPM and POm thalamic neurons. *Nat. Commun.* **15**, 5883 (2024).

31. C. Liberale, G. Cojoc, P. Candeloro, G. Das, F. Gentile, F. De Angelis, Micro-optics fabrication on top of optical fibers using two-photon lithography. *IEEE Photonics Technol. Lett.* **22**, 474–476 (2010).

32. A. Antonini, C. Liberale, T. Fellin, Fluorescent layers for characterization of sectioning microscopy with coverslip-uncorrected and water immersion objectives. *Opt. Express* **22**, 14293–14304 (2014).

33. X. Li, Y. Li, Y. Zhou, J. Wu, Z. Zhao, J. Fan, F. Deng, Z. Wu, G. Xiao, J. He, Y. Zhang, G. Zhang, X. Hu, X. Chen, Y. Zhang, H. Qiao, H. Xie, Y. Li, H. Wang, L. Fang, Q. Dai, Real-time denoising enables high-sensitivity fluorescence time-lapse imaging beyond the shot-noise limit. *Nat. Biotechnol.* **41**, 282–292 (2023).

34. F. Helmchen, M. S. Fee, D. W. Tank, W. Denk, A miniature head-mounted two-photon microscope. high-resolution brain imaging in freely moving animals. *Neuron* **31**, 903–912 (2001).

35. W. Göbel, J. N. D. Kerr, A. Nimmerjahn, F. Helmchen, Miniaturized two-photon microscope based on a flexible coherent fiber bundle and a gradient-index lens objective. *Opt. Lett.* **29**, 2521–2523 (2004).

36. C. J. Engelbrecht, R. S. Johnston, E. J. Seibel, F. Helmchen, Ultra-compact fiber-optic two-photon microscope for functional fluorescence imaging in vivo. *Opt. Express* **16**, 5556–5564 (2008).

37. N. Accanto, F. G. C. Blot, A. Lorca-Cámara, V. Zampini, F. Bui, C. Tourain, N. Badt, O. Katz, V. Emiliani, A flexible two-photon fiberscope for fast activity imaging and precise optogenetic photostimulation of neurons in freely moving mice. *Neuron* **111**, 176–189.e6 (2023).

38. F. G. C. Blot, D. Decombe, A. Lorca-Cámara, M. Anquetil, V. de Sars, C. Tourain, B. C. Forget, N. Accanto, V. Emiliani, 2P-FENDO-II: A fiber bundle microscope for all-optical, large field-of-view brain studies in freely moving mice. *Cell Rep. Methods* **6**, 101305 (2026).

39. J. B. Pawley, Ed., Handbook of Biological Confocal Microscopy (Springer, New York, NY, 2006).

40. Z. Cheng, Y. Li, J. Lin, M. Cui, Large field-of-view volumetric deep brain imaging through gradient-index lenses. *Nat. Commun.* **16**, 9465 (2025).

41. Y. Yang, Y. Jeon, Z. Dong, J. K. W. Yang, M. H. Moghaddam, D.-S. Kim, D. K. Oh, J. Lee, M. Hentschel, H. Giessen, D. Kang, G. Kim, T. Tanaka, Y. Zhao, J. Bürger, S. A. Maier, H. Ren, W. Jung, M. Choi, G. Bae, H. Chen, S. Jeon, J. Kim, E. Lee, H. Kang, Y. Park, D. D. Nguyen, I. Kim, P. Cencillo-Abad, D. Chanda, X. Jing, N. Liu, I. V. Martynenko, T. Liedl, Y. Kwak, J.-M. Nam, S.-M. Park, T. W. Odom, H.-E. Lee, R. M. Kim, K. T. Nam, H. Kwon, H.-H. Jeong, P. Fischer, J. Yoon, S.-H. Kim, S. Shim, D. Lee, L. A. Pérez, X. Qi, A. Mihi, H. Keum, M. Shim, S. Kim, H. Jang, Y. S. Jung, C. Rossner, T. A. F. König, A. Fery, Z. Li, K. Aydin, C. A. Mirkin, J. Seong, N. Jeon, Z. Xu, T. Gu, J. Hu, H. Kwon, H. Jung, H. Alijani, I. Aharonovich, J. Kim, J. Rho, Nanofabrication for nanophotonics. *ACS Nano* **19**, 12491–12605 (2025).

42. C. M. Benoit, D. A. Ganea, R. Paricio-Montesinos, J. Esser, C. Thome, J. M. Janssen, A. Sattin, S. M. Innocenti, S. Krabbe, A. Lüthi, T. Fellin, M. Engelhardt, J. Gründemann, Axon initial segment dynamics during associative fear learning. *Nat. Neurosci.* **29**, 535–542 (2026).

43. T. D. Pereira, N. Tabris, A. Matsliah, D. M. Turner, J. Li, S. Ravindranath, E. S. Papadoyannis, E. Normand, D. S. Deutsch, Z. Y. Wang, G. C. McKenzie-Smith, C. C. Mitelut, M. D. Castro, J. D'Uva, M. Kislin, D. H. Sanes, S. D. Kocher, S. S.-H. Wang, A. L. Falkner, J. W. Shaevitz, M. Murthy, SLEAP: A deep learning system for multi-animal pose tracking. *Nat. Methods* **19**, 486–495 (2022).

44. L. Sità, M. Brondi, R. P. Lagomarsino de Leon, S. Curreli, M. Panniello, D. Vecchia, T. Fellin, A deep-learning approach for online cell identification and trace extraction in functional two-photon calcium imaging. *Nat. Commun.* **13**, 1529 (2022).

45. A. Giovannucci, J. Friedrich, P. Gunn, J. Kalfon, B. L. Brown, S. A. Koay, J. Taxidis, F. Najafi, J. L. Gauthier, P. Zhou, B. S. Khakh, D. W. Tank, D. B. Chklovskii, E. A. Pnevmatikakis, CaImAn an open source tool for scalable calcium imaging data analysis. *eLife* **8**, e38173 (2019).

46. A. Finkelstein, D. Derdikman, A. Rubin, J. N. Foerster, L. Las, N. Ulanovsky, Three-dimensional head-direction coding in the bat brain. *Nature* **517**, 159–164 (2015).

47. J. M. Olson, K. Tongprasearth, D. A. Nitz, Subiculum neurons map the current axis of travel. *Nat. Neurosci.* **20**, 170–172 (2017).

48. Z. Zhang, S. J. Liu, B. Mattison, J. Muir, N. Spurr, C. K. Kim, W. Yang, High-speed neural imaging with multiplexed miniaturized two-photon microscopy. *Cell Rep. Methods* **5**, 101221 (2025).

49. C. Zhao, Y. Zhu, D. Zhang, Q. Fu, M. Pan, R. Wu, A. Wang, H. Cheng, Millimeter field-of-view miniature two-photon microscopy for brain imaging in freely moving mice. *Opt. Express* **31**, 32925–32934 (2023).

50. T. Slettmoen, N. L. de Jong, H. Eneqvist, E. R. Skytøen, W. Zong, M.-B. Moser, E. I. Moser, Place cells in CA1 lack topographical organization of firing locations. *Proc. Natl. Acad. Sci. U.S.A.* **123**, e2528601123 (2026).

51. R. Fink, S. Imai, N. Gockel, G. Lauer, K. Renken, J. Wietek, P. J. Lamothe-Molina, F. Fuhrmann, M. Mittag, T. Ziebarth, A. Canziani, M. Kubitschke, V. Kistmacher, A. Kretschmer, E. Sebastian, J. Ottens, D. Schmitz, T. Terai, J. Gründemann, S. I. Hassan, T. Patriarchi, A. Reiner, M. Fuhrmann, R. E. Campbell, O. A. Masseck, PinkyCaMP: An mScarlet-based calcium sensor with enhanced brightness, photostability and multiplexing capabilities. *Nat. Methods* **23**, 998–1010 (2026).

52. A. Klioutchnikov, D. J. Wallace, C. Berdahl, A. Sugi, J. Sawinski, J. N. D. Kerr, Simultaneous two- and three-photon multiplane imaging across cortical layers in freely moving mice. *Nat. Methods* **23**, 1437–1446 (2026).
